# Critical Fluctuations as an Early Warning Signal of Sports Injuries? A Proof of Concept Using Football Monitoring Data

**DOI:** 10.1186/s40798-024-00787-5

**Published:** 2024-12-16

**Authors:** Niklas D. Neumann, Jur J. Brauers, Nico W. van Yperen, Mees van der Linde, Koen A. P. M. Lemmink, Michel S. Brink, Fred Hasselman, Ruud J. R. den Hartigh

**Affiliations:** 1 Department of Psychology, Faculty of Behavioral and Social Sciences, University of Groningen, Groningen, The Netherlands; 2 Department of Human Movement Sciences, Faculty of Medical Sciences, University of Groningen, University Medical Center Groningen, Groningen, The Netherlands; 3 Football Club Groningen, Groningen, The Netherlands; 4 Behavioral Science Institute, Radboud University, Nijmegen, The Netherlands

**Keywords:** Football, Complex dynamic systems, Nonlinear time series analysis, Injury prediction, Dynamic complexity, Process monitoring, Multidisciplinarity, Personalized approach, Early warning signals, Critical fluctuations

## Abstract

**Background:**

There has been an increasing interest in the development and prevention of sports injuries from a complex dynamic systems perspective. From this perspective, injuries may occur following critical fluctuations in the psychophysiological state of an athlete. Our objective was to quantify these so-called Early Warning Signals (EWS) as a proof of concept to determine their explanatory performance for injuries. The sample consisted of 23 professional youth football (soccer) players. Self-reports of psychological and physiological factors as well as data from heart rate and GPS sensors were gathered on every training and match day over two competitive seasons, which resulted in an average of 339 observations per player (range = 155–430). We calculated the Dynamic Complexity (DC) index of these data, representing a metric of critical fluctuations. Next, we used this EWS to predict injuries (traumatic and overuse).

**Results:**

Results showed a significant peak of DC in 30% of the incurred injuries, in the six data points (roughly one and a half weeks) before the injury. The warning signal exhibited a specificity of 95%, that is, correctly classifying non-injury instances. We followed up on this promising result with additional calculations to account for the naturally imbalanced data (fewer injuries than non-injuries). The relatively low F_1_ we obtained (0.08) suggests that the model's overall ability to discriminate between injuries and non-injuries is rather poor, due to the high false positive rate.

**Conclusion:**

By detecting critical fluctuations preceding one-third of the injuries, this study provided support for the complex systems theory of injuries. Furthermore, it suggests that increasing critical fluctuations may be seen as an EWS on which practitioners can intervene. Yet, the relatively high false positive rate on the entire data set, including periods without injuries, suggests critical fluctuations may also precede transitions to other (e.g., stronger) states. Future research should therefore dig deeper into the meaning of critical fluctuations in the psychophysiological states of athletes.

**Key Points:**

- Complex Systems Theory suggests that sports injuries may be preceded by a warning signal characterized by a short window of increased critical fluctuations.
- Results of the current study showed such increased critical fluctuations before 30% of the injuries. Across the entire data set, we also found a considerable number of critical fluctuations that were not followed by an injury, suggesting that the warning signal may also precede transitions to other (e.g., healthier) states.
- Increased critical fluctuations may be interpreted as a window of opportunity for the practitioner to launch timely and targeted interventions, and researchers should dig deeper into the meaning of such fluctuations.

**Supplementary Information:**

The online version contains supplementary material available at 10.1186/s40798-024-00787-5.

## Background

Hardly any athlete finishes their career unscathed, as sports injuries are a ubiquitous and disturbing aspect of the athletic trajectory [1]. Those who are affected often experience performance decrements and suffer financially [2, 3]. Given the substantial incidence of injuries [4] and the harm they cause, the sports field would highly benefit from any kind of anticipation or prediction. In that way, practitioners may be supported in their decision-making on whether, for instance, the training plan needs to be adjusted for an athlete.

While the prediction of sports injuries is a subject of significant interest and value, it continues to pose a challenge even after many years of dedicated research. Obviously, injuries can never be predicted perfectly, but recent research suggests that strides can be made by accounting for the dynamic complexity through which injuries come about [5–7]. In the present empirical research, we will specifically target this dynamic complexity. To be more specific, as a proof of concept, we will apply an analytic strategy from the complex dynamic systems toolbox to detect warning signals, which can be an important avenue in the field of sports science and medicine.

### Past Approaches and Future Directions of Sports Injury Prediction

Previous research on sports injury prediction measured isolated or monodisciplinary risk factors at one or a few points in time, and analysed data with linear methods at the group level [8–12]. Prediction models performed rather low in forecasting injuries, they were poorly developed (i.e., not validated, no code provided, high risk of bias), and not applicable in practice [8, 13, 14]. Researchers have highlighted the importance of data quality, since poor or unsuitable data can lead to misleading results and hinder the ability to accurately predict outcomes, such as sports injuries [e.g., 15]. Further, much focus has been put on why injuries occur (i.e., revealing relevant factors) but the question of how those factors lead to injuries and when athletes are at increased risk is generally lacking.

As a response, researchers have suggested to study sports injuries from a complex dynamic systems perspective [5–7, 16–21]. Complex systems consist of factors that interact over time in a non-linear manner [5, 6]. Those factors coordinate their behaviour while generating patterns that are adapted to their environment. When a pattern is maintained by a system, the pattern can be called an attractor state, that is a state to which the system is “attracted” [22, 23]. Interestingly, systems may exhibit multistability, meaning they can exist in multiple stable states (e.g., different levels of health). This kind of relative stability indicates that a system may hop between two or more stable states.

**Fig. 1:**
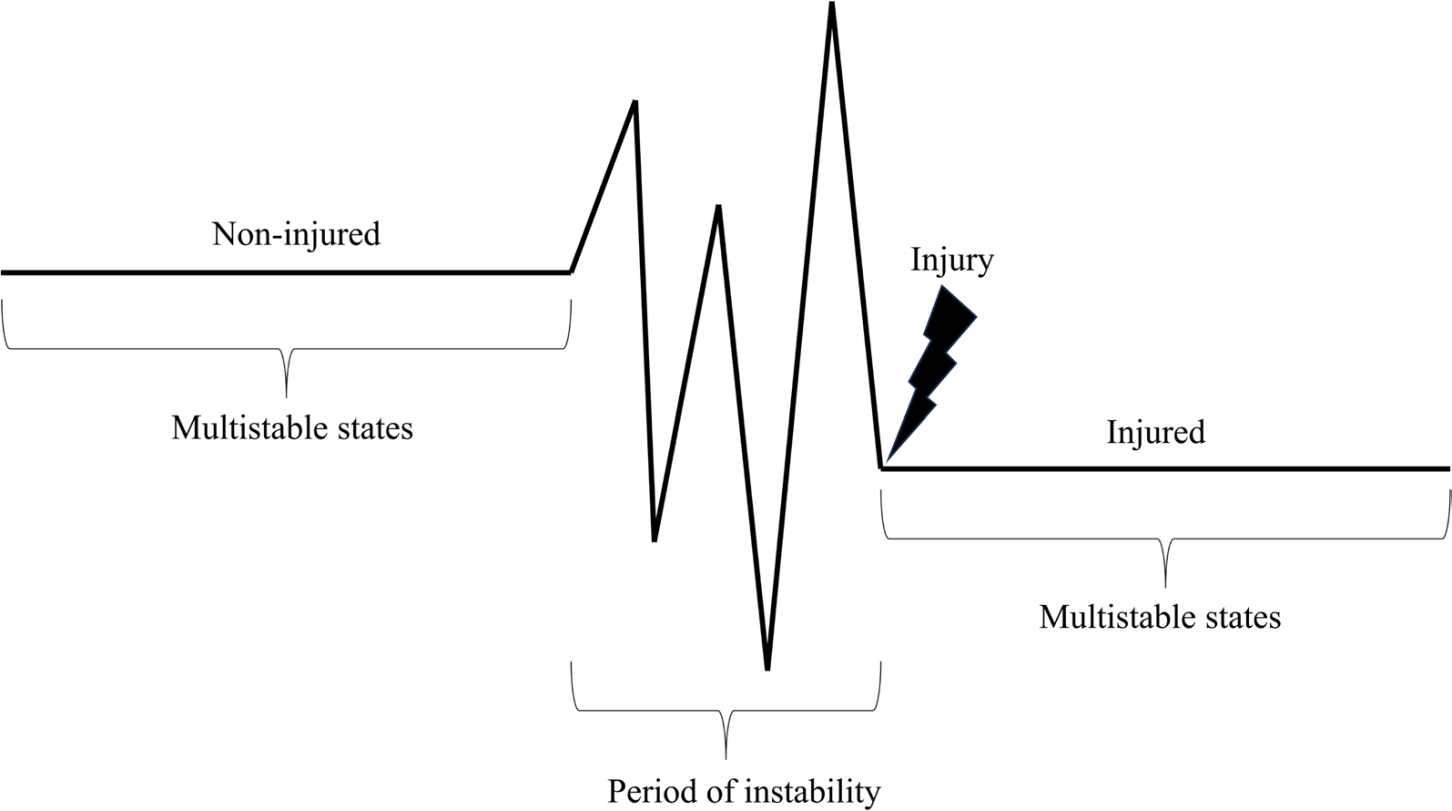
Theoretical illustration of critical fluctuations before an injury**.** The transition from the non-injured condition to the injured condition is often characterized by a period of instability, like critical fluctuations . This period can be considered an EWS. Note that in the non-injured and injured conditions, athletes can possess multiple stable states and transition between them

Complex systems typically have certain tipping points in which abrupt changes (phase transitions) from one attractor state to another can occur [5, 6, 20, 23–27]. An injury would be the result of this so-called phase transition, a system-wide reorganisation, from healthy states to injured states [6]. For such a transition to take place, the stability of the prevailing attractor state has become weakened [28]. Theory and empirical findings in the fields of ecology, financial markets, and psychology show that, in such a phase of instability, critical fluctuations can be observed as a relatively short window of increased variability and turbulence (for an illustration see Fig. 1) [6, 25, 28–33]. Such fluctuations are therefore called “Early Warning Signals” (EWS) and might be an explanation of how injuries occur. To be more specific, instead of analysing absolute values of screening tests or daily measures at the group level, which will likely not work in predicting injuries [12, 34], theory suggests that strides can be made by investigating the complex dynamics, and more specifically, critical fluctuations, at the individual level [6, 35]. Given the parallel between injury development and complex system dynamics, an interesting question is therefore whether such critical fluctuations can also be found before injuries.^1^.

### Non-Linear Time Series Analysis

To detect critical fluctuations, time series analysis methodologies are needed [e.g., 28–31,36,37]. A promising methodological tool in this regard is the *Dynamic Complexity* (DC) algorithm, which can detect heavy and irregular variability in a system’s behaviour [25, 29, 35, 38]. DC was developed for the real-time monitoring of human change processes and is therefore specifically suited for non-linear, non-stationary, and short and coarse-grained time series that are typical in psychology, sports science and medicine. So far, DC has already successfully been applied to investigate changes in mood of psychological disorders and physical activity. For instance, a study by Olthof and colleagues [29] showed that increased critical fluctuations predicted sudden shifts in mood in the next four days for patients receiving psychotherapy. In the same line, research on walking behaviour found a positive and significant association between increased critical fluctuations and a loss of step counts in the next few days [38].

Recently, Schiepek et al. [35] provided a first exploration of the potential merits of DC as an EWS of sports injuries. In their case study, one professional football (soccer) player filled out the sports process questionnaire across 77 days using an app-based system. During that time, he suffered a twisted cruciate ligament. Results showed that DC reached a peak of critical instability just before the injury. This is a promising finding, but on the basis of this case study, we cannot conclude that such a signal is specific for identifying injuries. Hence, more research is needed to confirm the potential and robustness of these results across athletes and injuries. Further, the study did not determine the time window for which the warning signal appears before the injury, and it is unclear how a peak was defined. In the current in-depth study of DC on sports injuries we address these limitations in Schiepek et al.’s [35] study and account for the complex multidisciplinarity of traumatic and overuse sports injuries.

### The Present Study

As a proof of concept, the present study aims to test the explanatory performance of critical fluctuations as an EWS for injuries in youth football players. To meet this aim, it is important to monitor psychological and physiological variables of individual players on a daily basis [9, 39–42]. Such monitoring can be realized via an online application and sensors, for instance [35, 39, 43].^2^ There is a myriad of factors that can be measured, yet, the decision on what to measure should be based on what prior scholarly work suggests may be precursors of sports injuries and what is realistic and feasible to collect from the sample. On the psychological level, this may include key factors in sports performance and injuries such as self-efficacy, motivation, and mood [35, 44–49]. In addition, athletes may regularly experience unenjoyable training sessions and bad performances [39]. On the physiological level, the recovery status may be monitored before every training session or competition [50–52]. Internal load factors such as the heart rate and the session rating of perceived exertion (sRPE), as well as external load factors such as sprints and total distance covered, are also typically related to injuries [18, 53–58].

In line with the complex dynamic systems theory, we hypothesized that increased critical fluctuations (i.e., cumulative peaks of DC) in relevant factors precede the occurrence of an injury. Last, the study intends to explore which measured factor(s) (i.e., psychological, physiological, self-reports, sensors) reveal the most peaks in destabilizations.

## Methods

### Subjects

We collected data from 55 youth male players of the U-18 and U-21 teams (16 to 20 years old) of a premier league (Eredivisie) football club in the Netherlands. Twenty-three of these players were included in the analysis because they fulfilled the inclusion criteria (more details under 2.3.1. Data Pre-Processing). Once a player started playing at the club, he was informed about the data collection process. By signing an informed consent, he could then decide whether he wanted to permit the use of his data for research purposes or not (for the present study, all players provided consent). The players competed in the highest national league of their age category. They had between six and eight training sessions per week, composed of two strength sessions and four to six field sessions of 60 to 75 min and 75 to 90 min, respectively, and matches on the weekend. Participants were familiar with strength training from age 12 on and strength training sessions took place around midday, after the field sessions. Due to personal data protection, further potentially identifiable information (e.g., height, weight, position, team, specific type of injury) is not reported.

### Design, Measures, and Procedure

For every training and match, up to two competitive seasons, we collected psychological and physiological data. To be more specific, players answered self-report questions on self-efficacy, motivation, mood, performance self-evaluation, enjoyment, session rating of perceived exertion (sRPE) and total quality of recovery (TQR) on a tablet computer near the locker room without staff or team members being present. All measures were part of the normal, daily team monitoring routine at the club. Polar TeamPro sensors (Polar Electro Oy, Kempele, Finland) were used to collect data on the duration, total distance covered, sprints, and heart rate for every training session and match (see Table 1).

**Table 1:** Data collection

Time of the day	Measured factor	Self-report question	Measurement scale	Origin of measurement
T1: In the morning up to 30 min before the first training session or match	Recovery	How good is your recovery?	CRS from 6 (*very, very poor recovery*) to 20 (*very, very good recovery*)	[e.g., 67,68]
	Self-efficacy	How confident are you that you can perform maximally today?	VAS from 0 (*not at all confident*) to 100 (*very confident*)	[e.g., 48,69]
	Motivation	How motivated are you to perform maximally today?	VAS from 0 (*not at all motivated*) to 100 (*maximally motivated*)	[e.g., 48,61]
	Mood	How much are you in the mood to train/play the match today?	VAS from 0 (*not at all in the mood*) to 100 (*very much in the mood*)	[e.g., 47,62]
T2: During the training session or match	Distance		Meter	[e.g., 18,53]
	Sprint		Number of sprints > 25 km/h (for the U-21) and > 19.8 km/h (for the U-18) per session^1^	[e.g., 53,56]
	Duration		Minutes	[e.g., 54,65]
	Heart rate in zone 5		Beats per minute; output is the number of seconds spent in zone 5 (i.e., 92–100% of an individual’s max heart rate)^2^	[e.g., 53,55]
T3: At the end of the day up to 30 min after the last training session or the match	Exertion	How hard was the training/match?	CRS from 6 (*very, very light*) to 20 (*very, very hard*)	[e.g., 55,70]
	Perceived performance	How well did you perform today?	VAS from 0 (*very bad (far below my capabilities*)) to 100 (maximally (*to the best of my capabilities*))	[e.g., 39,71]
	Enjoyment	How much did you enjoy the training session(s)/the match today?	VAS from 0 (*not at all*) to 100 (*very much*)	[e.g., 39,72]

*CRS* category-ratio scale, *T* time point, *VAS* visual analogue scale

^1^Researchers have argued that high-intensity zones can contribute to an increased risk of injuries, which is why we decided to look into the fluctuations of this zone [91]. However, note that this may also be a limitation because injuries may also emerge in lower speed zones.

^2^The maximal heart rate is based on the Interval Shuttle Run Test (ISRT), an intermittent fitness test [92]. Players performed the test before the start of the first and second half of the season, and the maximal heart rate was updated once the test was performed. This can also be a limitation, because the maximum heart rate may change non-linearly every session. Practically, however, it would not be feasible to perform an ISRT every session to determine the maximal heart rate. Alternatively, one may assess the heart rate variability for every session.

The players were familiarized with daily data collection since the age of 15 or since their enrolment in the academy [15]. Monitoring is a key component of the clubs' philosophy and is integral to the individual development of their players. At the beginning of each season, and at several points throughout the season, coaches emphasized the importance of this practice: to foster individual development, enhance performance, and reduce the likelihood of injuries. This approach helps to address common limitations of subjective self-reports, such as socially desirable responses, response fatigue, and compliance issues [59].

The single-item self-reports are either validated questions (recovery, exertion) or questions that previous research has used and proposed to tailor to one’s context (self-efficacy, motivation, mood, perceived performance, and enjoyment; see Table 1 for references). While the use of single items is justified to reduce time, costs, and participant burden, and to allow for repeated administration before athlete performance [60–62], we acknowledge that single-item measures may have limitations in capturing the full complexity of certain constructs. However, the literature supports their use, as the added benefit of multiple items is modest in most cases [60, 63]. The RPE consists of one item (“How hard was the training/competition?”) and is a subjective estimate of the psychological and physiological stress imposed on the athlete [64]. Later, we multiplied the RPE score by the duration of the training session to obtain a measure for the internal training load for the analysis, the session RPE (sRPE) [65]. If there were two load scores a day, which occasionally happened after two different types of training, we added them up to capture the daily load [66]. Finally, next to the questions explained in the table, the team physician recorded injury occurrence (yes vs. no), and injury-related time-loss (in days), including the mechanism (i.e., traumatic or overuse) [56].

### Data Set and Statistical Analysis

The analysis was performed with R and RStudio [73]. The R code has been made publicly available (see Code Availability).

#### Data Pre-Processing

The original data set consisted of 55 players from two youth teams. The following criteria were established to include players for the analysis: At least one injury occurrence was reported of a player between measurement seven and the last seven measurements (see 2.3.3 Proof of Concept for more background on this inclusion criteria) and a player did not miss more than 20% of the values per measured factor.^3^ This resulted in a final sample of 23 players, yielding an average of 339 observations per player (range = 155–430). First, we imputed missing values with the R package *mice* [74]. As a next step, we normalized the data in order to be able to analyse and compare the results. To be more specific, self-report data from the Visual Analogue Scale was divided by 100 (i.e., the maximum), self-report data from the Category-Ratio Scale was divided by 20 (i.e., the maximum), and data from sensors was divided by the maximum value of each factor of every individual.

#### Early Warning Signals (EWS) Calculation

We calculated critical fluctuations of the self-report and sensor data with the R package *casnet* [75], using the Dynamic Complexity (DC) algorithm. Mathematically, DC is a multiplication of the degree of fluctuation (F) of a time series and the distribution (D) of values between the theoretical minimum and maximum of a scale [for validation and details see 25]. F is sensitive to the amplitude and frequencies of a time series and D to the scattering of values. Both measures were calculated within a moving window of seven data points and a window step of one, in order to identify non-stationary changes.^4^ In that way, the DC algorithm inherently considers the temporal structure and repeated measurements in the time series data. Next, we checked for cumulative complexity peaks (CCPs), which indicate whether the number of simultaneous peaks in DC of the time series was significant. A CCP is determined by conducting a one-sided z-test (one side, because we are looking for *increased* critical fluctuations) to check for significantly increased scores, which results in a new time series that mirrors the number of significant peaks on a specific day. Another one-sided z-test is performed on this new time series to determine whether this number is significant. Since critical fluctuations are expected to be present just before a transition, we tested which time window between one and ten data points prior to the injury would be optimal for explaining how injuries come about. We increased the window size in a step-by-step manner starting with one. When increasing the window size did not provide better results, the previous window was considered as the optimum.

#### Proof of Concept

We tested the explanatory performance of the warning signal as a proof of concept in four steps. First, we calculated the sensitivity of the DC analysis for every individual. The sensitivity evaluates the warning signal's effectiveness in correctly identifying injury cases, highlighting its ability to accurately detect true positive cases and emphasizing its explanatory performance in identifying injuries [76]. It is the ratio of the true positive rate (i.e., a significant CCP and the athlete was injured) to the true positive rate plus the false negative rate (i.e., no significant CCP and the athlete was injured). Second, we calculated the specificity of the DC analysis for every individual. The specificity assesses the accuracy of the warning signal to correctly identify true negative cases [76]. It is the ratio of the true negative rate (i.e., no significant CCP and the athlete was not injured) to the true negative rate plus the false positive rate (i.e., a significant CCP and the athlete was not injured). Third, we calculated the accuracy of the warning signal. Accuracy is simply the percentage of correct predictions (injury and non-injury) made by the model [76]. It is calculated by dividing the number of correct predictions by the total number of predictions made. The result is multiplied by 100.

The inherently imbalanced nature of the dataset in this research, with a strong bias towards the majority class (non-injuries), may result in less stable and reliable estimates of sensitivity, specificity, and accuracy. For this reason, we also calculated an F_1_ score (see formula 1) as the fourth and last step, by using the harmonic mean of precision and recall [77]:

1 $$  F_{1}  = \frac{{2\;*\;{\text{precision}}\;*\;{\text{recall}}}}{{{\text{precision}} + {\text{recall}}}}  $$

Precision is the ratio of the true positive rate (i.e., a significant CCP and the athlete was injured) to the true positive rate plus the false positive rate (i.e., a significant CCP and the athlete was not injured). Recall is another term for sensitivity. The F_1_ score falls between 0 and 1, with 0 indicating no explanatory power and 1 indicating perfect explanatory power. Important to note, however, is that this analysis takes place at the group level since all individual observations must be combined and treated as one large observation.

We applied the following rules for the analysis: If at least one warning signal occurred in the (to-be-determined) window before an injury, we counted it as one true positive signal (the remaining five data points were left out for the analysis because a warning signal appeared already, even if they were positive as well). If no warning signal appeared, we counted it as one false negative signal (and not six). Likewise, injury periods were not taken into account for the analysis, because the injury already happened, and turbulence can be expected in this period. Last, the first six data points were subtracted from each individual's time series for the calculation of positive and negative signals, because we used a seven-day overlapping moving window, which means that the analysis started on day seven.

In our final step, we determined which variable(s) show the most CCPs before an injury.

## Results

**Fig. 2:**
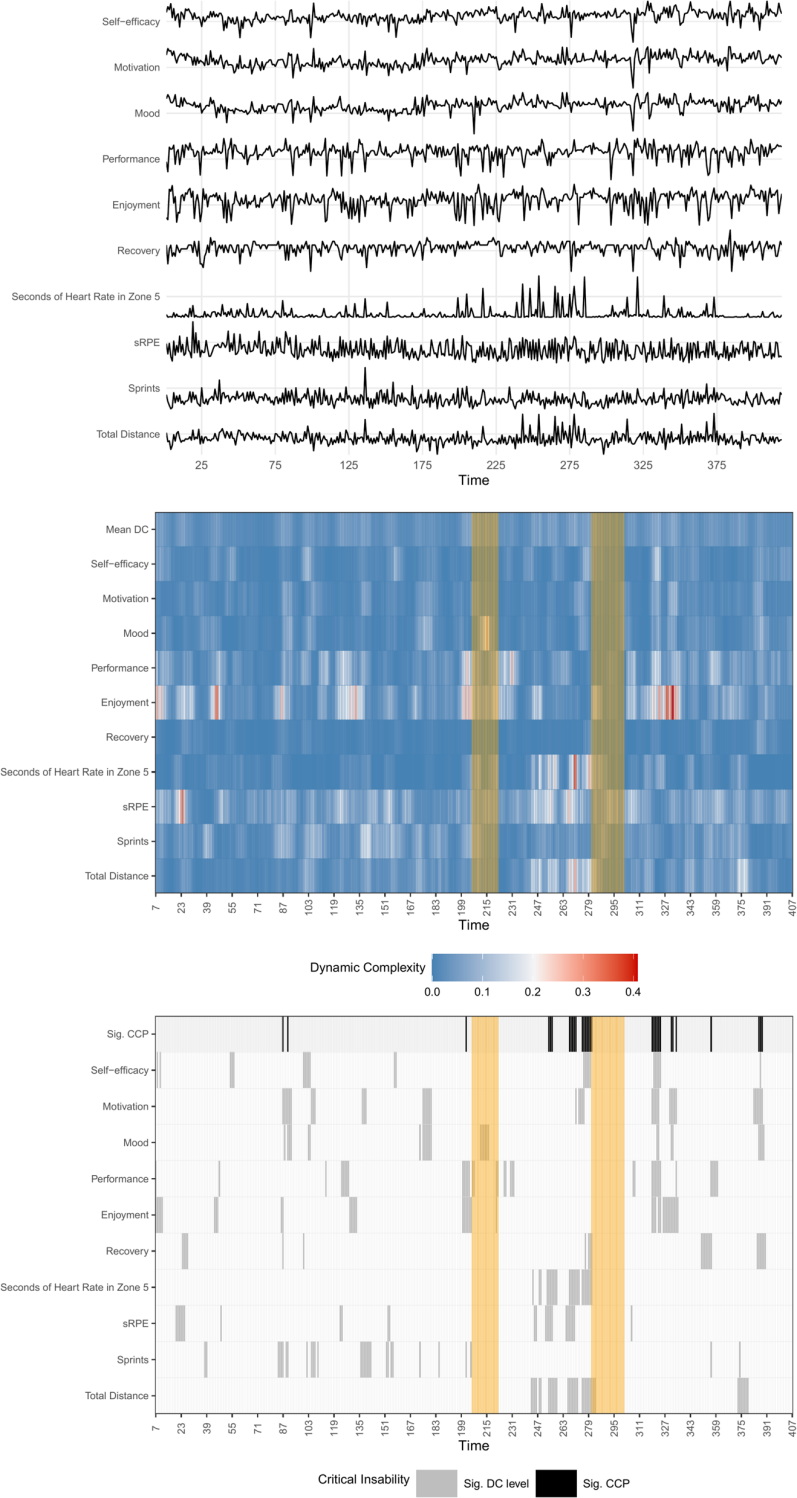
Dynamic Complexity Results of Player 9. CCP = Cumulative Complexity Peak, DC = Dynamic Complexity, sRPE = session Rating of Perceived Exertion. Multivariate raw time series (top figure), complexity resonance diagram (middle figure) and critical instability plot (bottom figure) of Player 9 suffering two injuries (injury period marked in orange/vertically shaded from top to bottom). The x-axis displays the number of data points. The y-axis shows each measured factor with the raw time series (top), the Dynamic Complexity values (middle), and the significant Dynamic Complexity values (bottom). The higher the Dynamic Complexity, the whiter and redder the plot becomes (middle figure). Significant Dynamic Complexity levels are colored grey, whereas significant Cumulative Complexity Peaks are colored black (bottom figure)

Players were injured 2.8 times on average across the measurement period (range = 1–6; total = 64). To illustrate, Fig. 2 shows the multivariate raw data, the complexity resonance diagram, and the critical instability plot of one representative player (Player 9; for the plots of the other players, see online resource). Note that for both injuries, a significant CCP appeared in the six data points before – an EWS.

The sensitivity analysis across all players showed that cumulative complexity peaks appeared for 30% (19 out of 64) of the injuries within six data points before their occurrence (range: 0–100%).^5^ The analysis further demonstrates that the warning signal exhibits a specificity of 95% (range = 86–100%). The accuracy reached a value of 94%. All three metrics were calculated per individual and then averaged across all individuals (individual-level analysis) [78]. Table 2 shows the confusion matrix from which these metrics were calculated and Table 3 provides more detailed information on injuries and the DC analysis for all individual players. Finally, the calculation of the F_1_ score, conducted at the group level, revealed an estimate of 0.08. This indicates poor performance in terms of both precision and recall, suggesting that the model's ability to correctly identify positive instances and avoid false positives is very low.

**Table 2:**
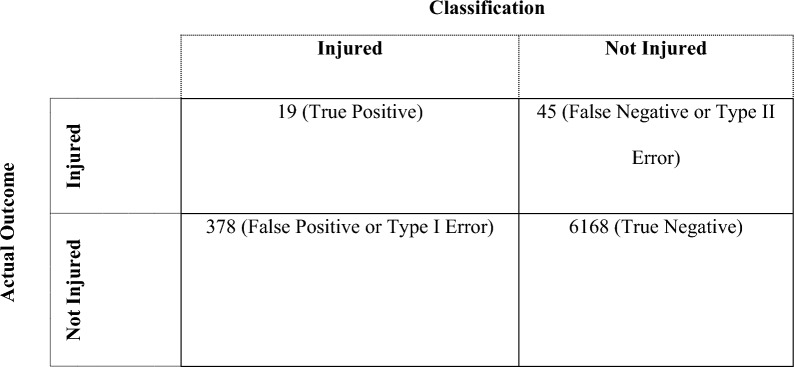
Confusion matrix showing the classification and actual outcome

**Table 3:** Descriptive statistics and explanatory performance of the DC analysis of all individual players

Player	Number of measurements	Number of injuries	Time-loss in days	Mechanism	Sensitivity	Specificity
1	176	1	12	T	0%	95%
2	427	2	22	T	50%	93%
			8	T		
3	248	4	3	T	50%	94%
			38	T		
			6	T		
			6	O		
4	351	2	5	T	50%	95%
			286	NA		
5	419	2	8	T	0%	96%
			12	T		
6	413	3	3	T	33%	95%
			13	O		
			35	T		
7	241	2	25	T	50%	97%
			37	T		
8	398	4	12	T	0%	97%
			30	T		
			17	T		
			25	O		
9	419	2	20	O	100%	94%
			26	T		
10	421	6	11	O	17%	95%
			9	O		
			22	O		
			28	T		
			8	T		
			51	T		
11	413	1	16	T	0%	95%
12	229	2	7	T	0%	94%
			5	T		
13	408	2	6	O	50%	86%
			22	T		
14	288	5	14	T	20%	95%
			27	T		
			12	O		
			10	T		
			271	T		
15	422	3	8	T	33%	94%
			21	O		
			24	T		
16	155	4	9	T	0%	100%
			15	T		
			6	T		
			62	T		
17	418	2	14	O	100%	95%
			23	O		
18	415	4	14	T	50%	97%
			23	O		
			22	T		
			4	T		
19	258	4	32	T	25%	99%
			5	T		
			13	T		
			20	T		
20	430	4	19	T	25%	91%
			64	O		
			26	NA		
			41	O		
21	423	3	29	T	33%	95%
			3	T		
			5	T		
22	168	1	2	T	0%	94%
23	247	1	4	T	0%	92%

Injuries that occurred before data point seven and in the last seven data points are left out (n = 3), because the window size of seven data points only allows to capture Dynamic Complexity changes in between. Time-loss in days refers to the lost time in actual days, not days of measurement

*NA* Not Available, *O* Overuse, *T* Traumatic

Finally, we visually inspected all critical instability plots (see online resource) to determine which measured factor(s) reveal the most peaks in destabilization: The factor sprint appeared most often in the Cumulative Complexity Peaks that correctly predicted an injury, that is, thirteen times. One injury could be predicted with psychological factors only (psychological self-reports), another two injuries with physiological factors only (physiological self-reports and sensor data), four injuries with only self-reports (i.e., psychological and physiological; no sensor data), and two injuries with sensor data only. In the 10 remaining injuries, a combination of the factors was found.

## Discussion

The present study tested if critical fluctuations can serve as an EWS of sports injuries by applying a tool from the complex dynamic systems toolbox – specifically the DC algorithm – to sports monitoring data. In doing so, we collected longitudinal data on psychological and physiological factors of 23 football players across two competitive seasons. The outcomes of the study are twofold. As hypothesized, the results show that critical fluctuations appear quite often before injuries. Theoretically speaking, this supports the notion that injuries may come about through a non-linear dynamic process and that the methodological toolbox of complex dynamic systems could provide such insights [5, 35]. Also, the findings are in line with DC research on mood and physical activity [29, 38], and specifically, the potential promising DC application to injuries as presented by Schiepek et al. [35].

Second, we took additional measures to check the robustness and practical usefulness of our results. More specifically, the warning signal exhibits a sensitivity of 30% and a specificity of 95%. This means that the model can correctly predict 30% of injury- (19 of 64) and 95% of non-injury instances, respectively. The accuracy revealed an estimate of 94%, meaning that it correctly predicts injuries and non-injuries 94% of the time. These results suggest good explanatory performance of the warning signal. However, we also controlled for the imbalance in the data by calculating an F_1_ score (at the group level). The result revealed an estimate of 0.08, which suggests that the model's overall ability to discriminate between injuries and non-injuries is rather poor, especially given the high false positive rate. In the section Practical Implications*,* we put these results into perspective by explaining what this would mean for the practitioner.

Given the discrepancy between the results based on two different analytic strategies, we strongly recommend including additional checks for future research as well. So far, studies on EWS before critical transitions have not tested this concept extensively. More specifically, studies outside the sports context often only study periods before transitions instead of the whole time series, which does not capture the warning signals’ actual explanatory (or predictive) performance. Furthermore, previous work tended to provide only one or two metrics (e.g., sensitivity and/or specificity) and did not consider the imbalance in the dataset. This could have led to an overly optimistic report of the predictive performance of EWS metrics and their relevance for practice. With regard to the current study, while critical fluctuations can signal an increased likelihood of injuries, such a warning signal did not always lead to an injury, which can be due to different reasons. For instance, the system can also return to the previous attractor after destabilization, meaning that it does not result in a phase transition. Given that the data in this study is collected as part of the club's everyday routine, where practitioners utilize the data to adjust training processes, it is possible that injuries were prevented without our or the practitioner’s awareness. Destabilization, therefore, could better be considered a window of opportunity, meaning that it provides information that the system is currently out of equilibrium and re-organizes itself [29]. In the same line, critical fluctuations can be seen as a general indicator of instability and not a predictor of a specific kind of order transition. This means that critical fluctuations may also precede other kinds of transitions (e.g., a performance loss or gain, a mental dip).

An interesting additional result is that the physiological sensor factor *sprint* appears most often in the CCPs, suggesting that they might play an important role in explaining how injuries come about. However, the results also show that for one injury, only psychological factors appeared, whereas for most other injuries, a combination of the factors (psychological and physiological, self-report and sensor data) is decisive. These outcomes highlight the need for a multidisciplinary approach, that is the integration of the more objective physiological data with the more subjective psychological perceptions, tailored to individual athletes [e.g., 39,79]. In the next sections, we discuss future directions for research and practical implications of the results for the sports field.

### Recommendations for Future Research

To the best of our knowledge, the present study is the first proof of concept of the DC algorithm as a potential EWS of sports injuries in a sample of football players. From a complex dynamic systems perspective, the DC algorithm can be considered a strong method since it does not have any specific statistical or parametric assumptions about the data, it mirrors increased complexity and noise of system dynamics before a phase transition, it can model linear and non-linear phenomena, and it can be used in real-time and not only for post data collection. The study itself has high ecological validity because measuring took place in the field as a daily routine and not in an experimental setting.

In accordance with the high false positive rate on the full time series data, previous research has shown that destabilization is not necessarily related to negative outcomes, such as injuries or increases in symptom severity. Indeed, it may also precede decreases in symptom severity, that is, the transition to a healthier state [22, 29]. Hence, future research should test whether destabilization in athletes’ attractors can also predict, for instance, performance gains or decreases in physical complaints. Ideally, this would feature testing the true predictive performance on new and unseen data.

It must be noted that complex systems theory does not suggest all injuries take place via abrupt transitions preceded by critical fluctuations, they may also develop more gradually and within the same state of attraction. To investigate the latter possibilities, researchers may study increases in network connectivity through inter-item correlation of recurrence networks for the detection of critical slowing down, indicating increased signs of synchronization before an injury [28, 35, 80]. A clustering algorithm can then provide a deeper understanding of the networks by creating phase profiles. Such profiles reveal the nature of a state that recurs (i.e., the combination of values for certain variables) and their stability (i.e., how often they appear and when they change to another state). Forthcoming studies may thereby further exploit the complex dynamic systems toolbox to detect dissimilar dynamics preceding critical transitions (e.g., through recurrence quantification analysis and recurrence plots) [28, 35]. Another interesting feature of complexity is hysteresis, the history-dependent behaviour of a system after a perturbation [81]. In the present study, we investigated whether injuries are preceded by increases in critical fluctuations, and hysteresis would suggest that the path (e.g., whether the training load was previously high or low) affects the likelihood of injury, even if the current load is the same.

Further, in this study, we used variables identified by previous research as key factors related to injuries. A promising direction would be to explore variables which may potentially be more adequate for future EWS studies. Specifically, research could focus on identifying so-called order parameters (i.e., collective variables), which are unique to the individual and context, representing the collective behaviour of the system being studied [23]. For example, in endurance exercises, heart rate variability (HRV) has been proposed as an order parameter reflecting a measure of fatigue [83]. In the context of this research, this would involve identifying subjective and objectively quantifiable psychophysiological order parameters [84].

Last, in our study, we merely relied on intensive monitoring data collected daily across a season. However, as Hecksteden et al. [85] proposed, the integration of screening tests (i.e., risk factors and protective factors) with monitoring data may be a promising avenue for sports injury forecasting (see Additional file 1).

### Practical Implications

In a perfect world, practitioners would always be correctly informed by an EWS about an upcoming injury. However, the world is too complex to capture all the information needed to make such reliable forecasts. The findings from our study indicate that, on average, an injury is quite often preceded by critical fluctuations, but at the same time false positives regularly occur. In practice, and based on the data we analysed, this would mean that the warning signal suggests to intervene 21 times per player on average over the course of two seasons, while one of these signals actually leads to an injury which can be prevented ((TP + FP)/TP). Likewise, the warning signal fails to suggest an intervention in 45 instances, which ultimately lead to an injury (two injuries on average per player; FN/number of players). While a high false negative rate may be more harmful than a high false positive rate, intervening 21 times may still appear extensive. However, it is important to recognize that interventions do not always need to be resource-intensive in terms of time and cost. They may simply be short verbal interactions between the (multidisciplinary) staff and the player. For such verbal interactions, practitioners may specifically address those variables that showed increased critical fluctuations in the DC analysis. For the first injury of Player 9 in Fig. 2 this concerns his performance, enjoyment, and sprints. In that way, practitioners are provided with a conversation starter and information on which knob to turn for which athlete.^6^

However, if implemented, such a warning system should be considered a decision-support tool for practitioners to adapt training processes and implement interventions instead of a replacement of the coach [86, 87]. In a recent study by Hecksteden et al. [88], it was emphasized that a blend of practitioner experience, judgment, and scientific evidence should be used for decision-making in sports. Without this balance, there is a risk of over-reliance on technology and the disengagement of applied sports scientists [89].

### Conclusion

The results of this study suggest that critical fluctuations often precede sports injuries, thereby supporting the complex dynamic systems perspective on injuries. At the same time, it cannot (yet) be considered a valid EWS for the real-time prediction of injuries in sports practice. Critical fluctuations might be interpreted as a window of opportunity because turbulence indicates re-organization of the system which is currently out of equilibrium. Practitioners may make use of this window by planning timely and targeted interventions to guide athletes toward desirable conditions. Future research should dig deeper into the meaning of critical fluctuations in the psychophysiological states of athletes.

## Supplementary Information

**Additional file 1**. Dynamic complexity plots of all palyers and more recommendations for future research.

## Data Availability

The data contains sensitive information about the subjects and is therefore restricted from openly sharing it. Requests can be made by researchers affiliated with universities or independent, non-commercial research institutes via DataVerseNL at https://dataverse.nl/dataset.xhtml?persistentId=doi:10.34894/KRRCNO&faces-redirect=true. The R code is openly accessible via the same link.

## Abbreviations

DC: Dynamic Complexity
EWS: Early Warning Signal
sRPE: Session Rating of Perceived Exertion
TQR: Total Quality of Recovery
CCP: Cumulative Complexity Peak

## Footnotes

1 Other features of complexity, not the focus of the present study, may also indicate instability and serve as Early Warning Signals. One such feature is critical slowing down, that is, the increasing time to return to equilibrium, which reflects a rigid, less flexible and less variant system that is losing resilience [29, 33].

2 Previous research has demonstrated that self-reports and sensor data reveal complex dynamics [38, 90]. Therefore, we can assume to actually capture and study them under these methods.

3 Missing data is inevitable in sports monitoring. In consultation with the football club, we decided that a maximum of 20% missingness per variable is still acceptable for the analysis (this refers to roughly one day per week on average). Data was mainly missing, because players forgot to fill out the questionnaire or the sensors did not work. However, the analysis showed that the data was not missing completely at random, which is why we performed multiple imputation (R package *mice*) on the data. Recently, it was shown that imputing missing data (preferably using multiple imputation methods) is important to avoid biases and to make valid data-driven decisions in sports [93].

4 The window size of seven data points refers to roughly one and a half weeks in the present context (note that the football club did not measure every day of the week but for every training or match day). Previous studies used the same (relatively short) window size [28, 29, 38]. A validation study has shown that short window sizes still report sufficiently good outcomes [25].

5 The analysis indicated that a six-data point window before injuries was optimal since extending the window by an additional data point did not improve the explanatory performance.

6 The approach in the present study involved using the same set of questions for all individuals. However, recent research suggests that personalized, individually meaningful questions can uncover crucial information about transitions that might otherwise remain hidden. Testing this approach in the sports field could be highly interesting and relevant [94].
