# Supplementary material for: Critical Fluctuations as an Early Warning Signal of Sports Injuries? A Proof of Concept Using Football Monitoring Data
Source: Sports Med Open. 2024 Dec 16;10:129. doi: 10.1186/s40798-024-00787-5 (PMC11649608; doi:10.1186/s40798-024-00787-5)
Supplement: Supplementary file 1 — Additional file 1. Dynamic complexity plots of all palyers and more recommendations for future research. [file 40798_2024_787_MOESM1_ESM.pdf]

Supplementary Information

Article Title: Critical Fluctuations as an Early Warning Signal of Sports Injuries? A Proof of Concept Using Football Monitoring Data

Journal: Sports Medicine - Open

Authors: Niklas. D. Neumann[a*], Jur J. Brauers[b], Nico W. Van Yperen[a], Mees van der Linde[c], Koen A.P.M. Lemmink[b], Michel S. Brink[b], Fred Hasselman[d], Ruud J.R. Den Hartigh[a]

Affiliations: [a]Department of Psychology, Faculty of Behavioral and Social Sciences, University of Groningen, Groningen, The Netherlands; [b]Department of Human Movement Sciences, Faculty of Medical Sciences, University of Groningen, University Medical Center Groningen, Groningen, The Netherlands; [c]Football club Groningen, Groningen, The Netherlands; [d]Behavioral Science Institute, Radboud University, Nijmegen, The Netherlands

Contact of the corresponding author: n.d.neumann@rug.nl

The figures below show the multivariate raw time series (top figure), complexity resonance diagram (middle figure) and critical instability plot (bottom figure) of all 23 players (injury period marked in orange/vertically shaded from top to bottom). The x-axis displays the number of data points. The y-axis shows each measured factor with the raw time series (top), the Dynamic Complexity values (middle), and the significant Dynamic Complexity values (bottom). Note that the time scale starts at data point seven because we used a seven-data point overlapping moving window to calculate the Dynamic Complexity. This means that the first available Dynamic Complexity value is at data point seven. The higher the Dynamic Complexity, the whiter and redder the plot becomes (middle figure). Significant Dynamic Complexity levels are coloured grey, whereas significant Cumulative Complexity Peaks are coloured black (bottom figure). CCP = Cumulative Complexity Peak, DC = Dynamic Complexity, sRPE = Session Rating of Perceived Exertion.

Supplementary Figure 1

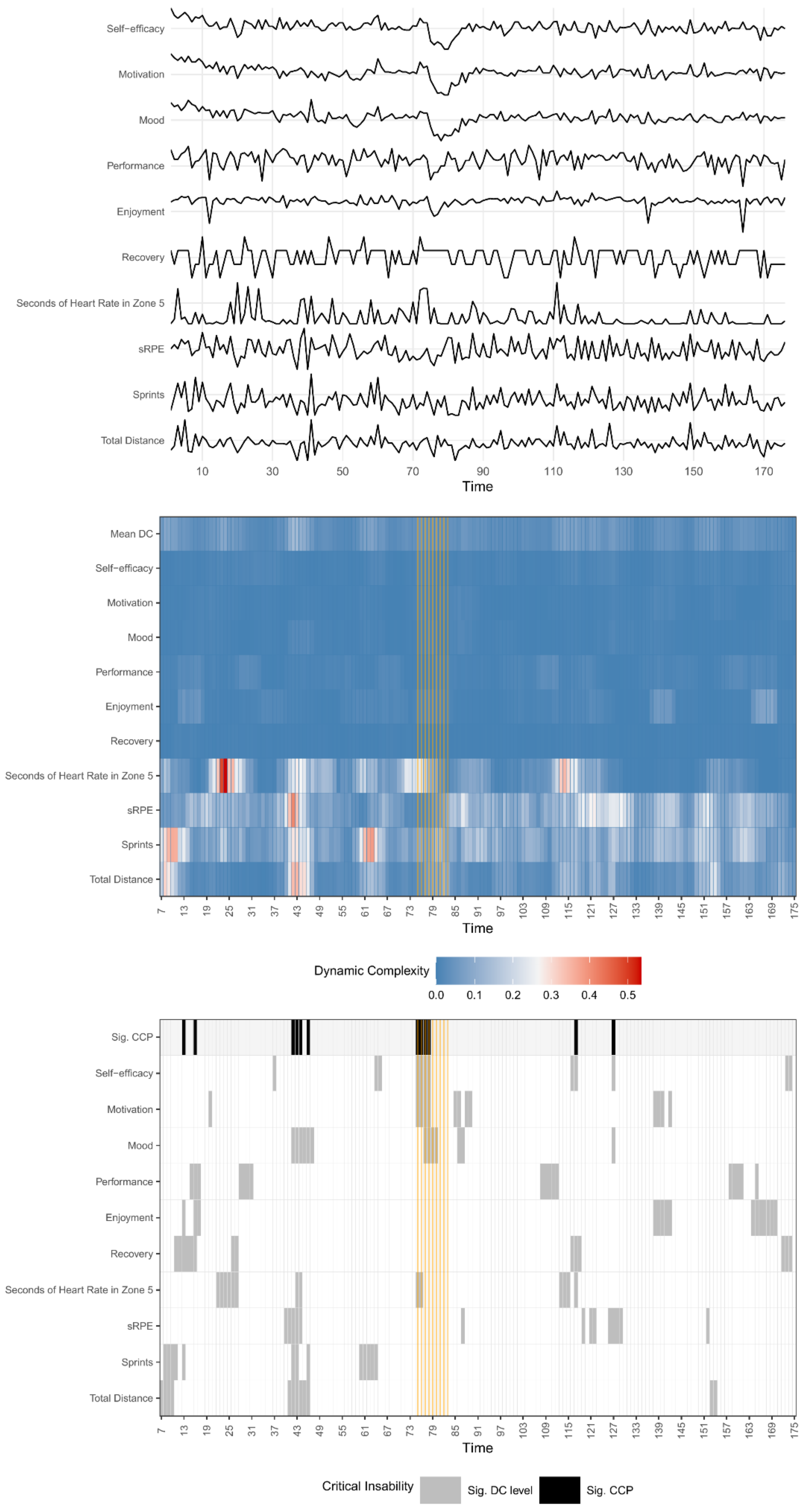

Supplementary Figure 2

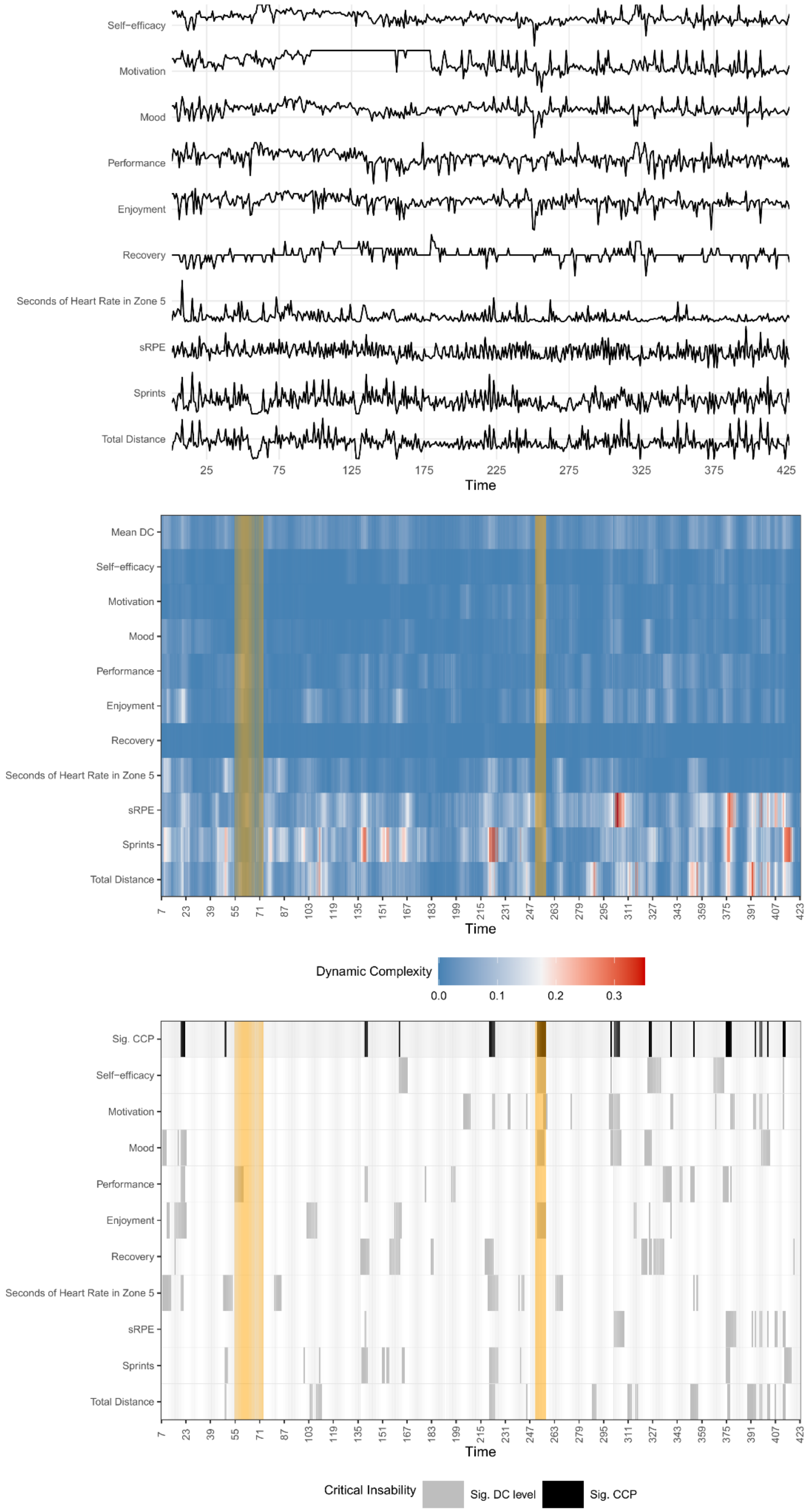

Supplementary Figure 3

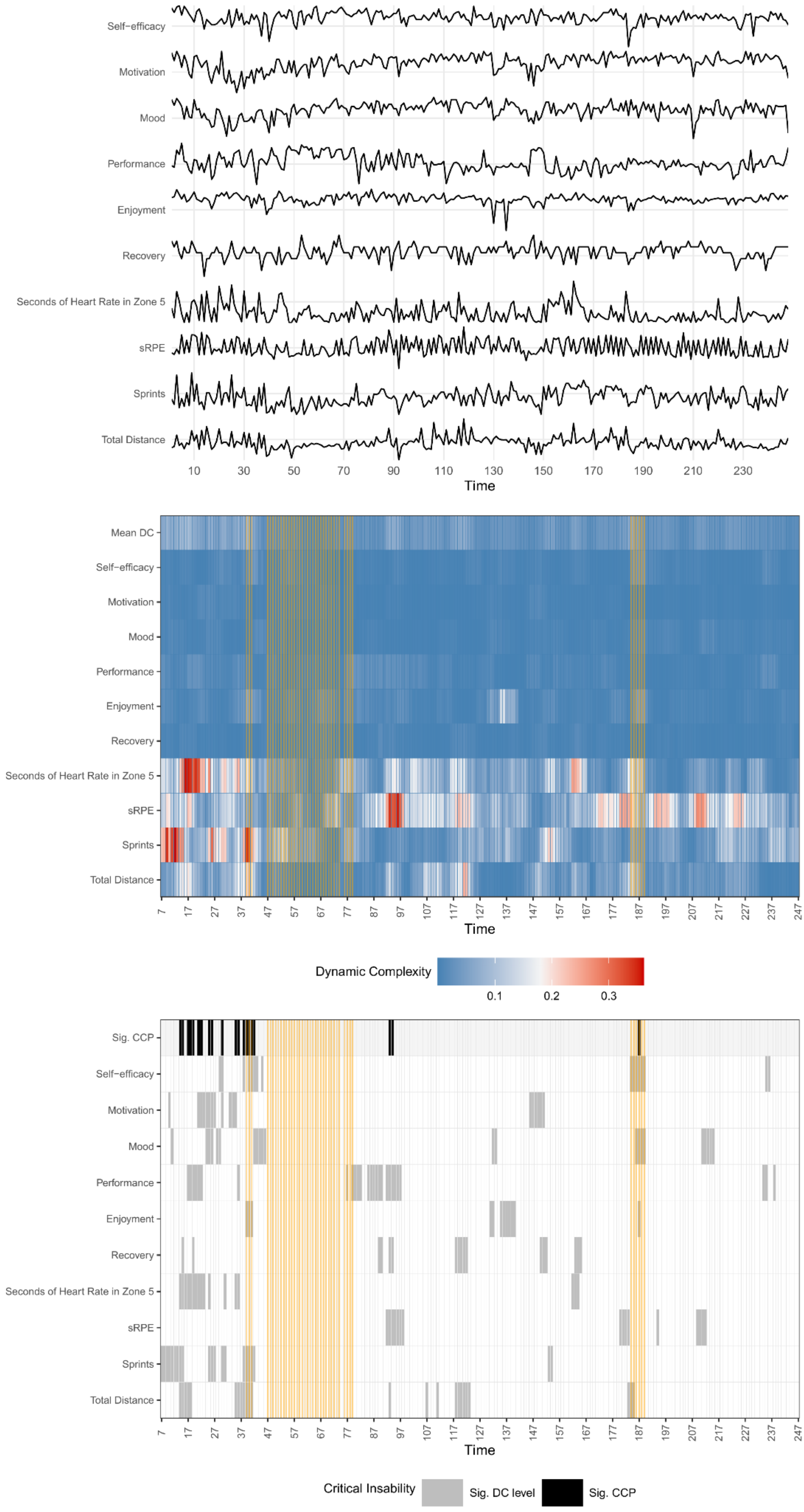

Supplementary Figure 4

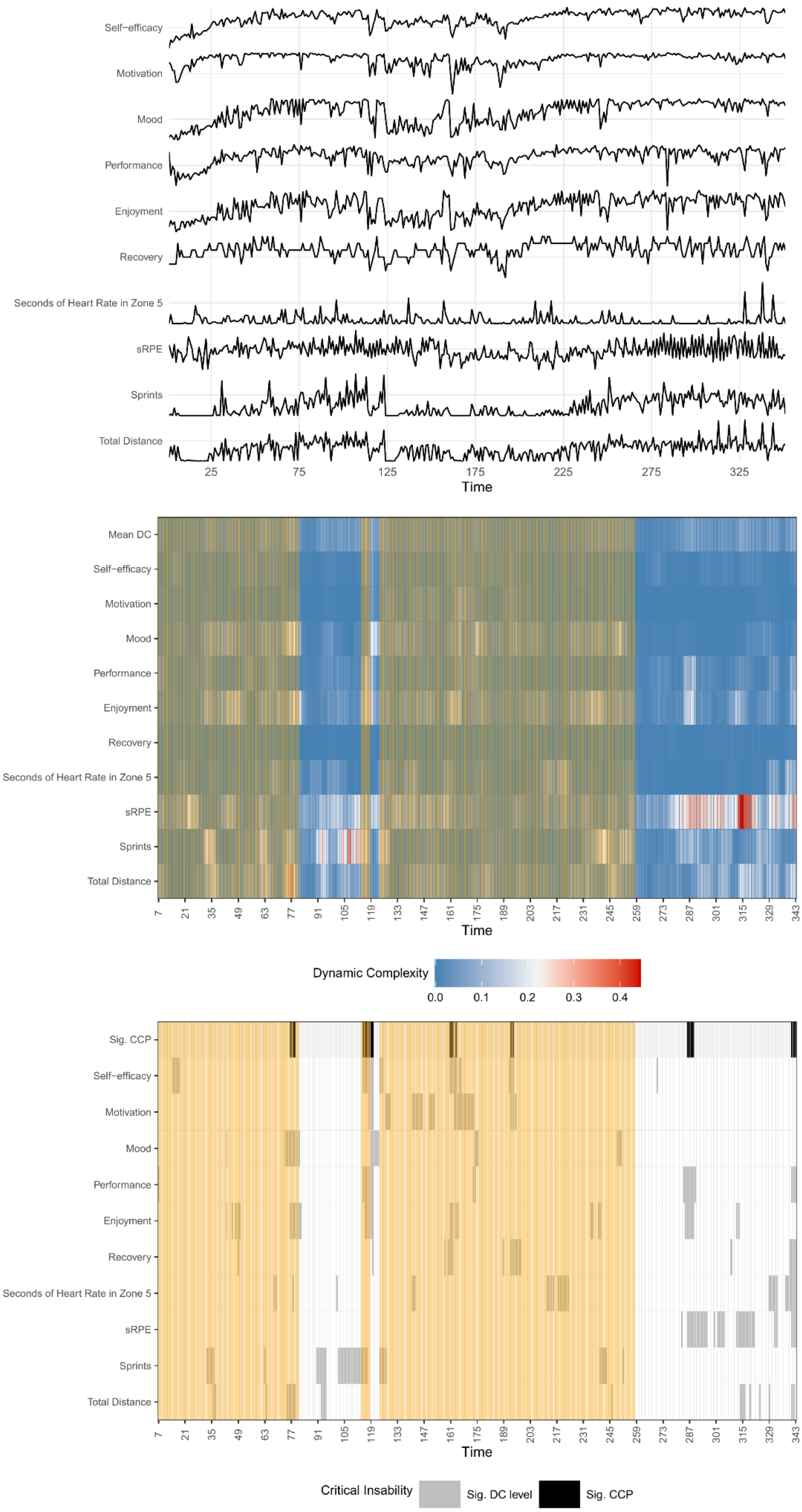

Supplementary Figure 5

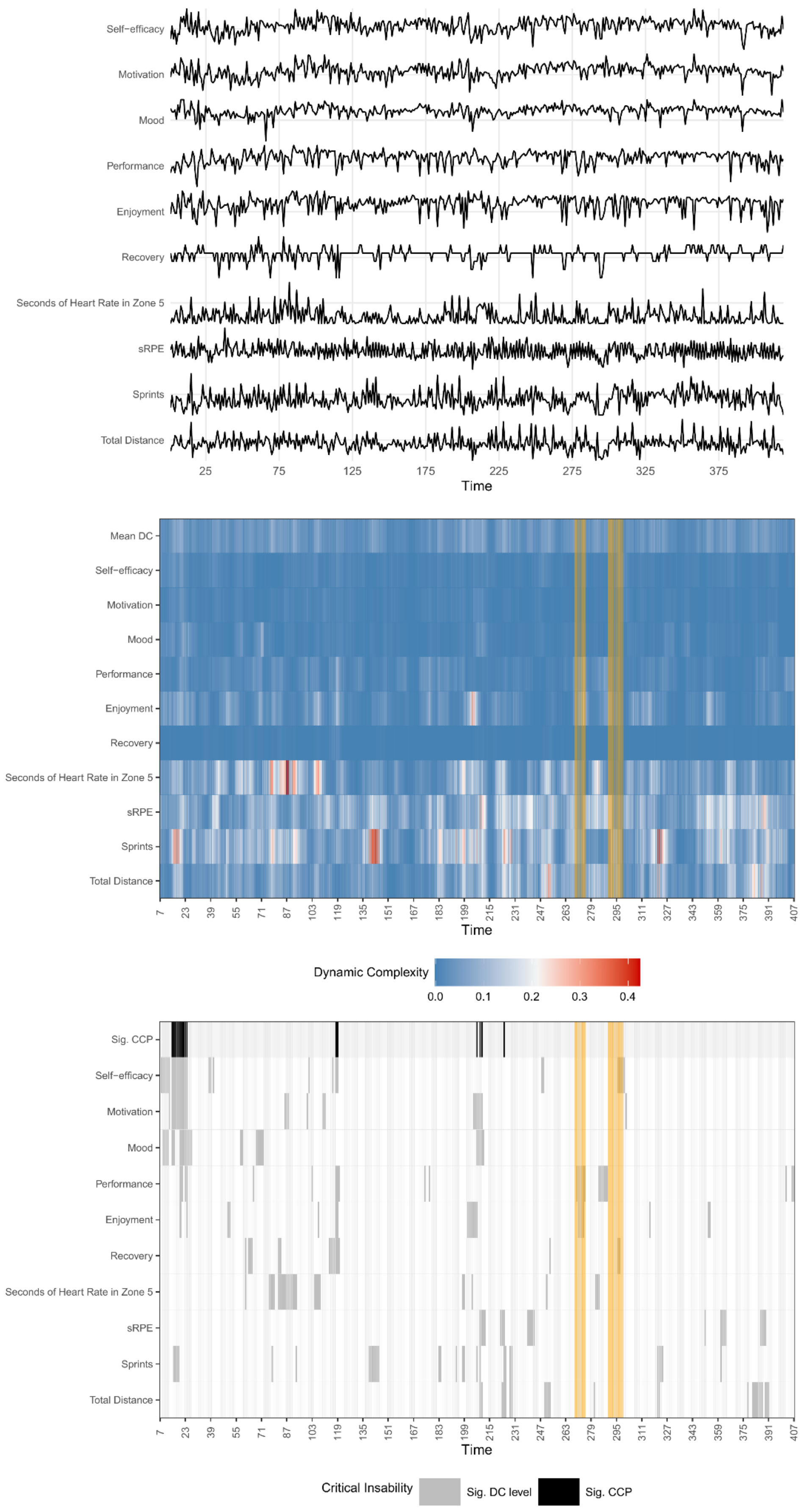

Supplementary Figure 6

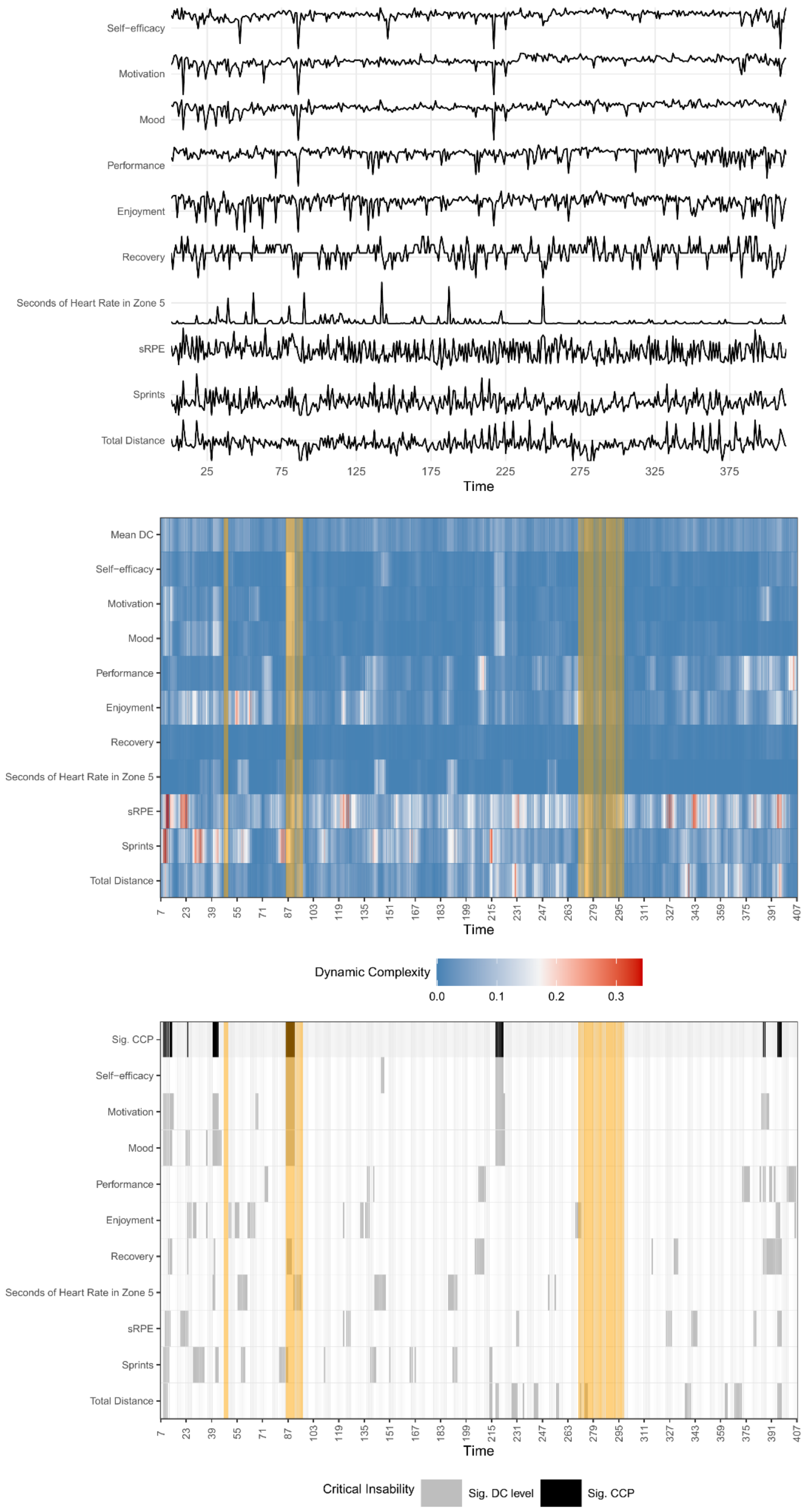

Supplementary Figure 7

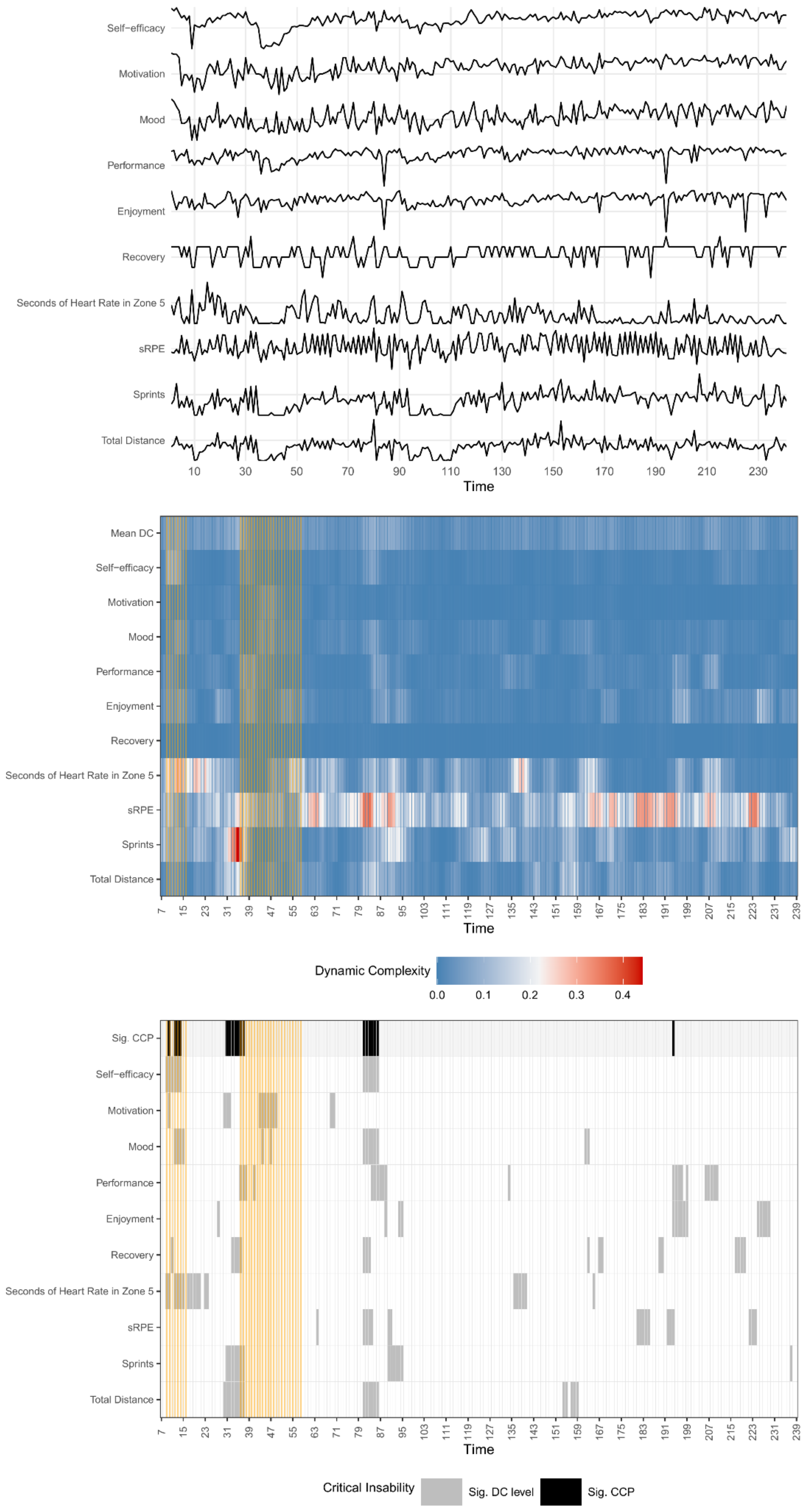

Supplementary Figure 8

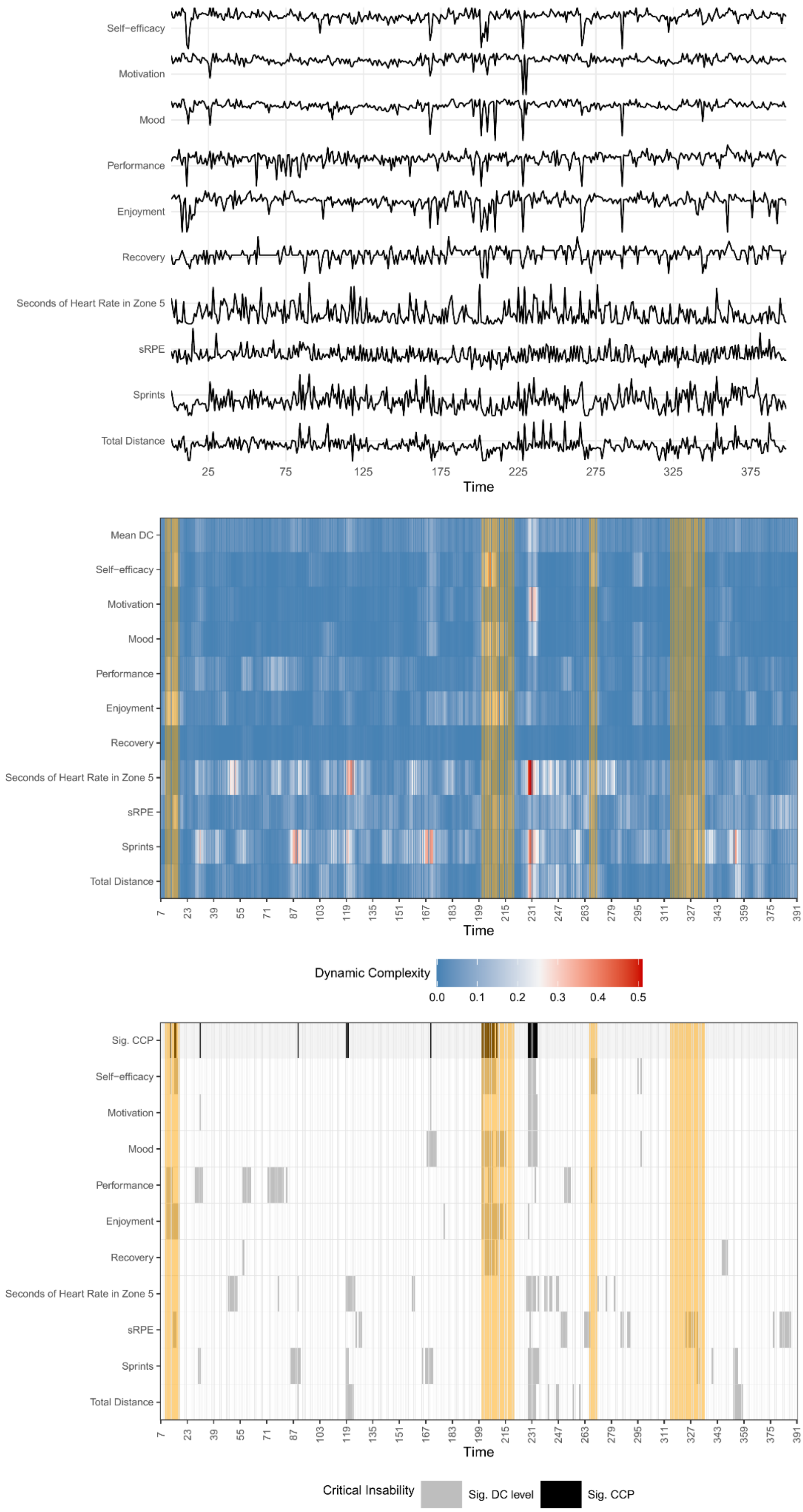

Supplementary Figure 9

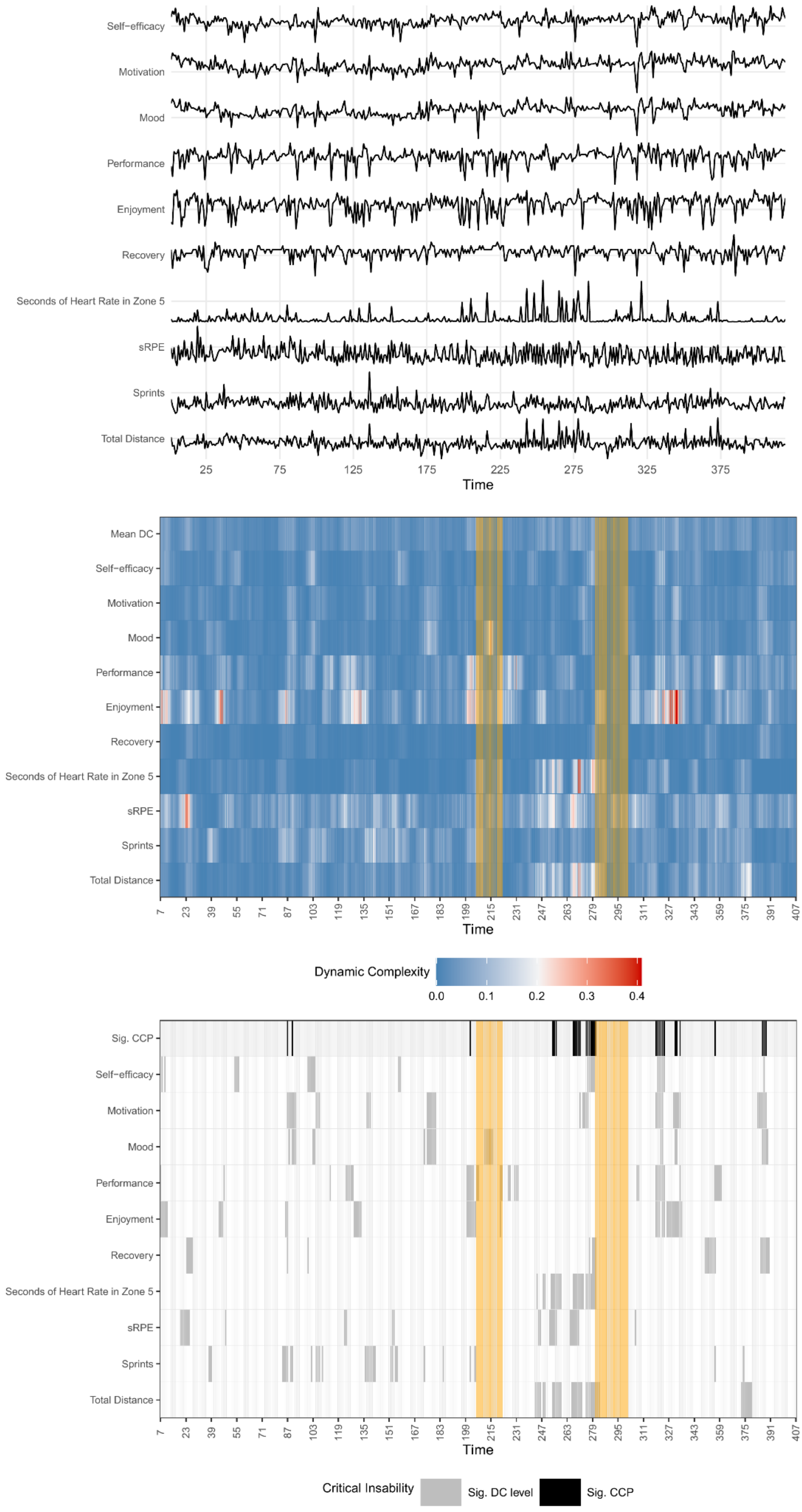

Supplementary Figure 10

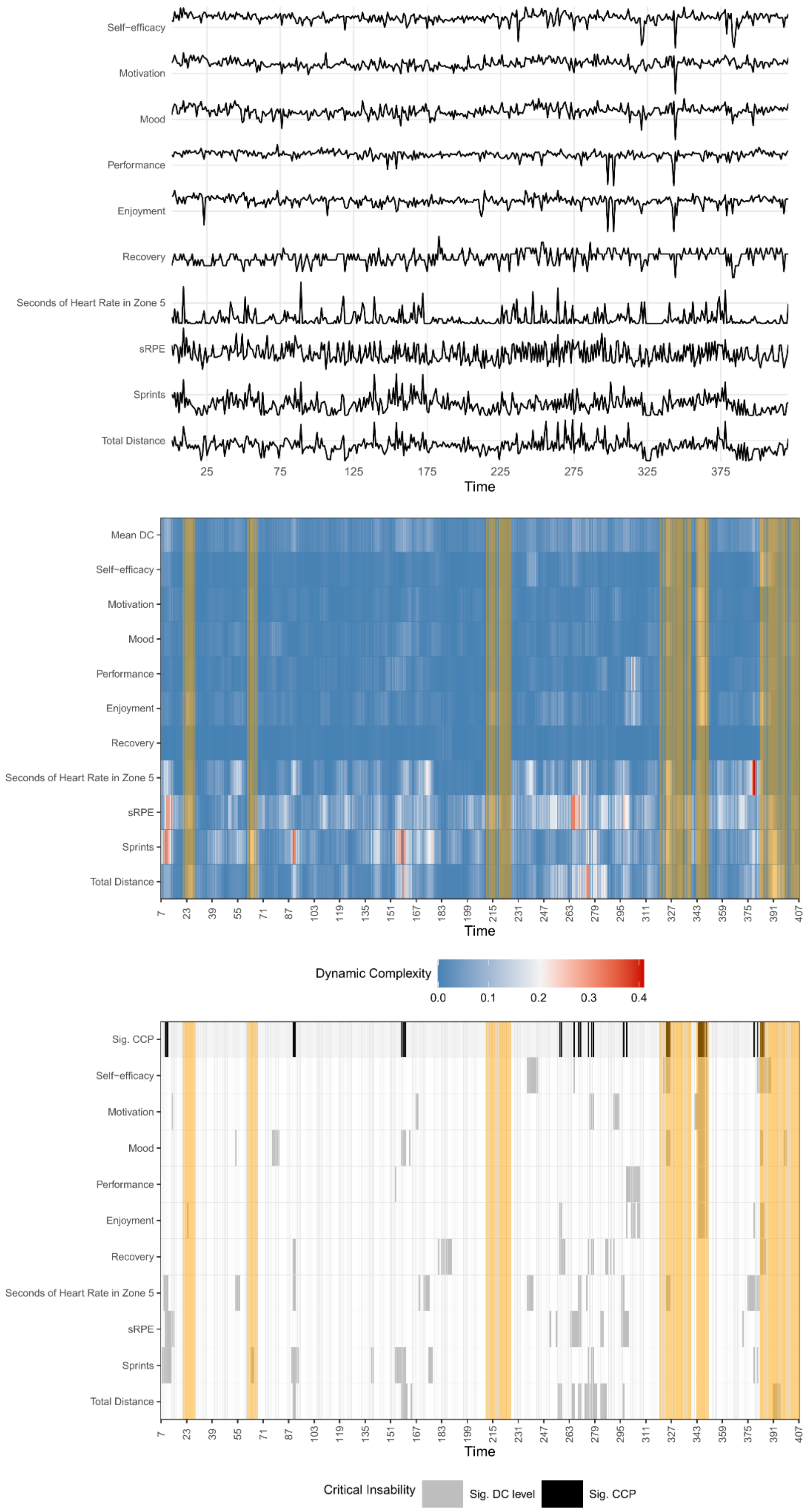

Supplementary Figure 11

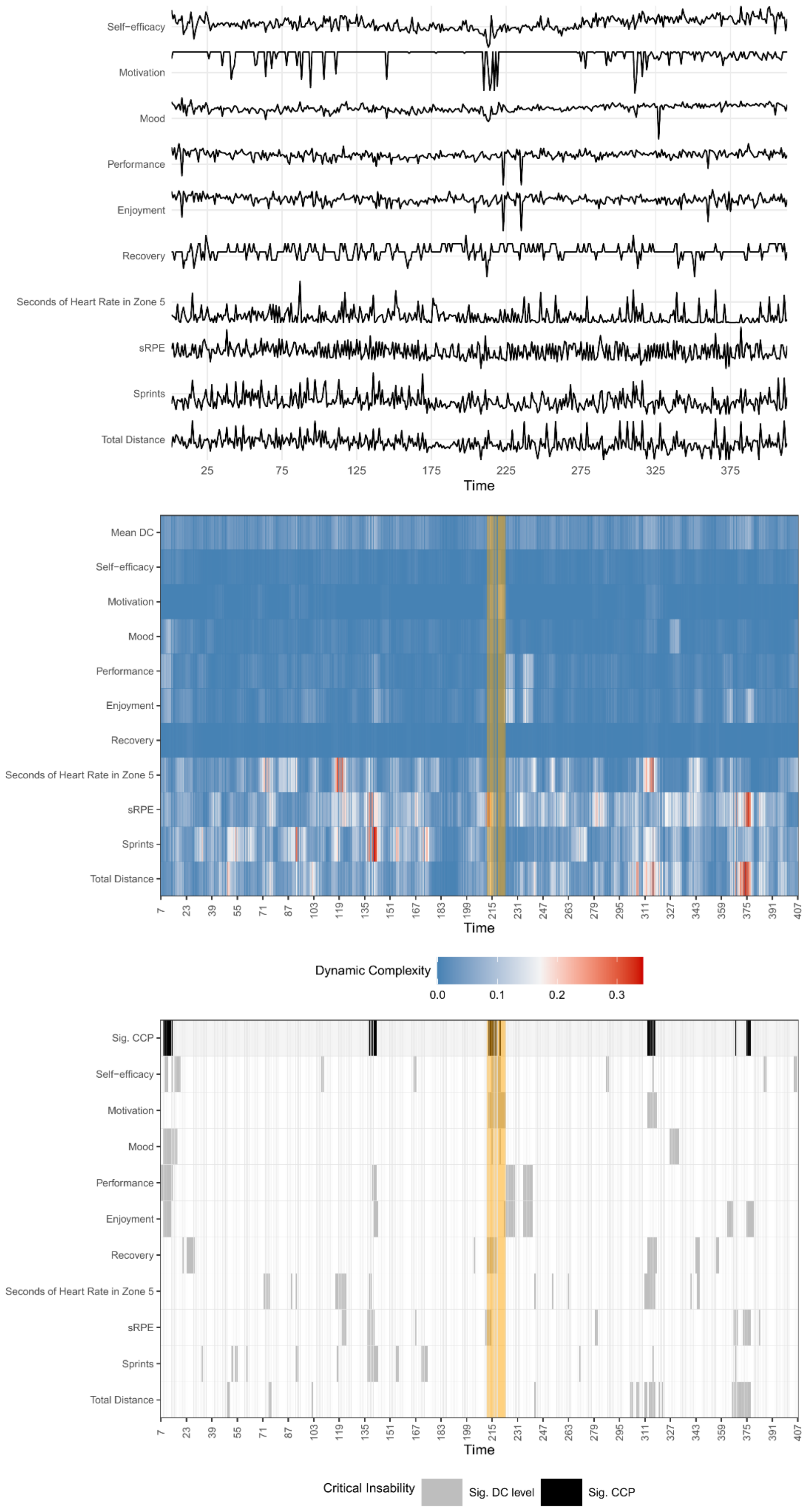

Supplementary Figure 12

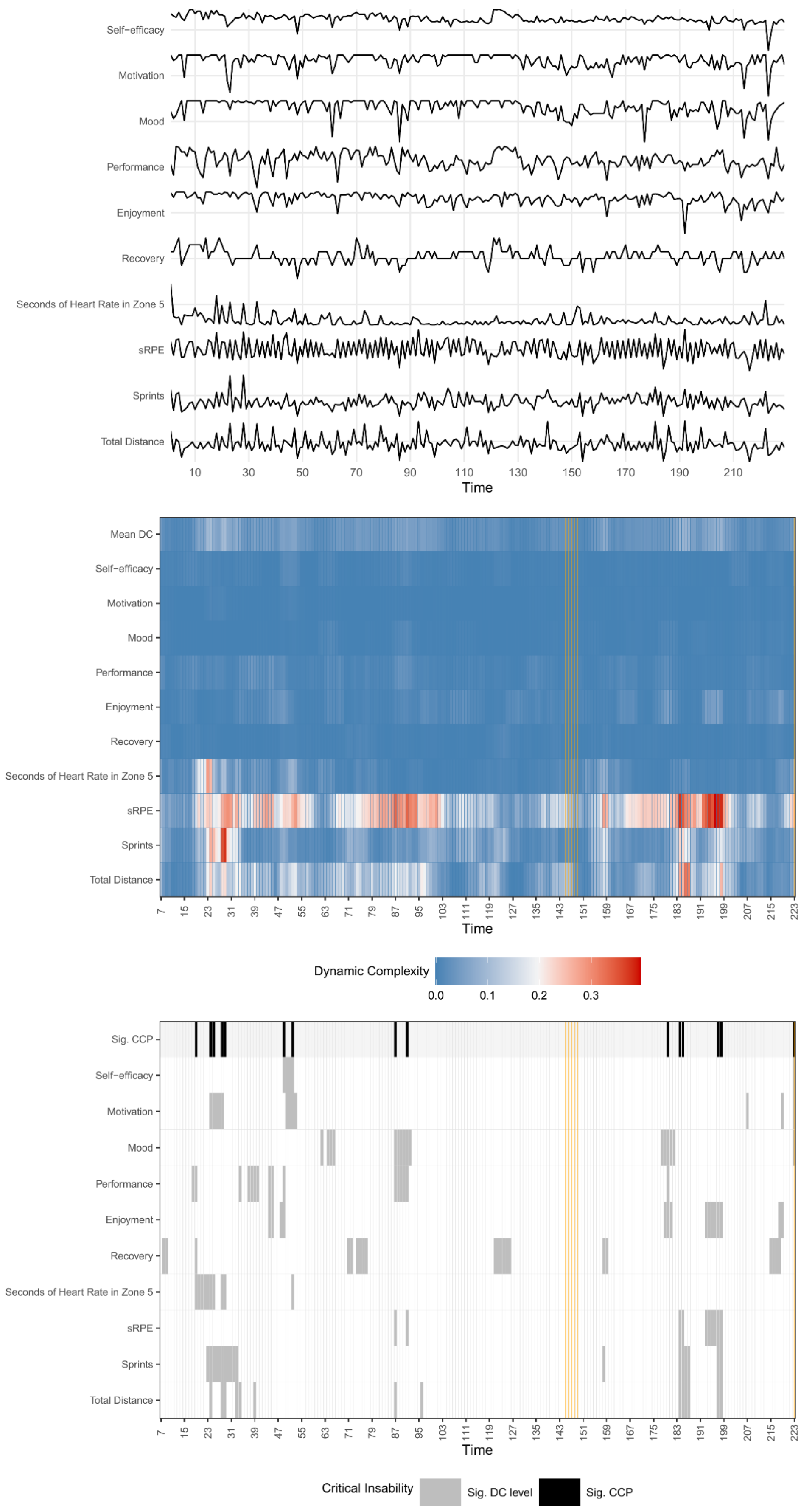

Supplementary Figure 13

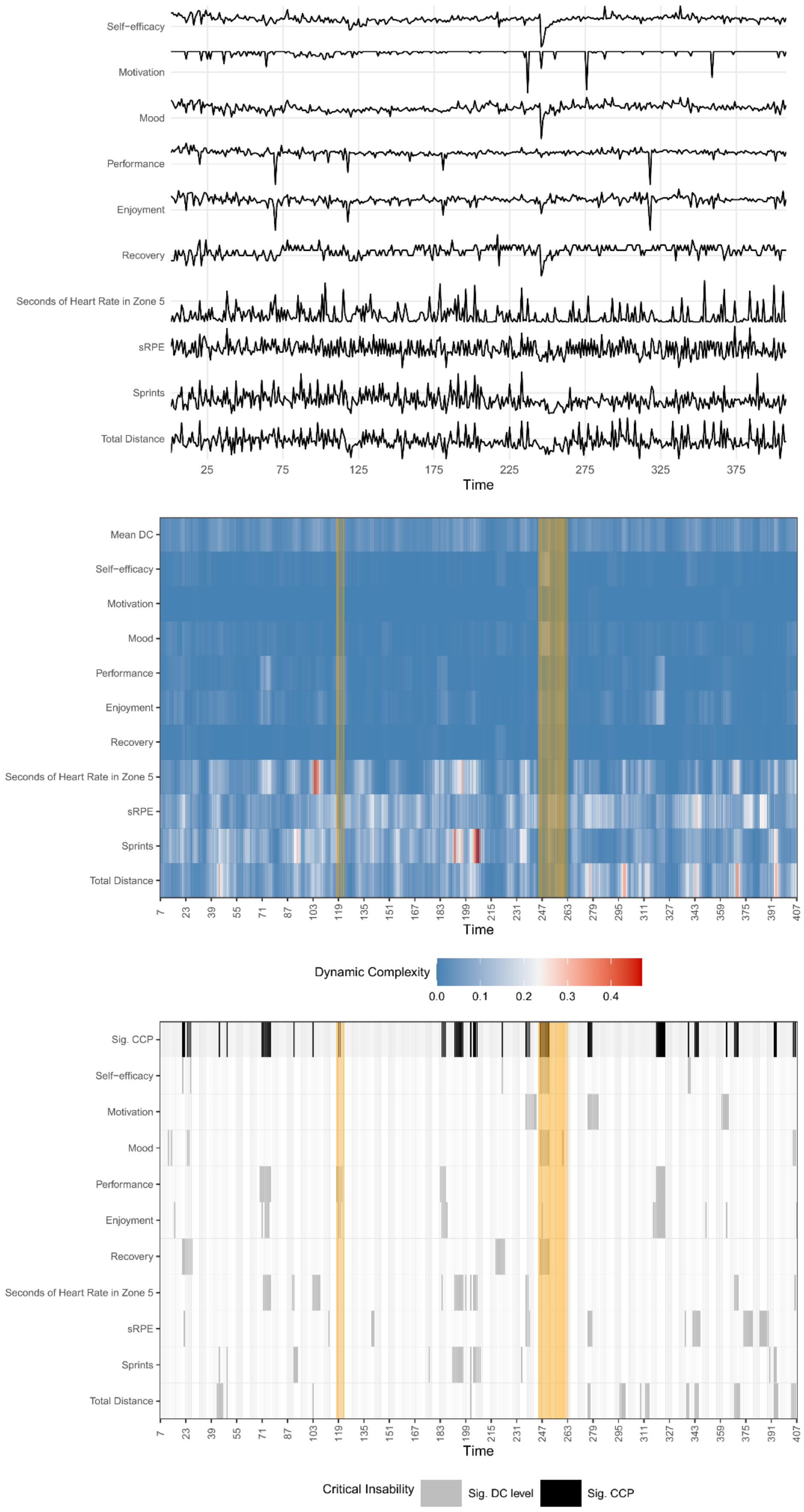

Supplementary Figure 14

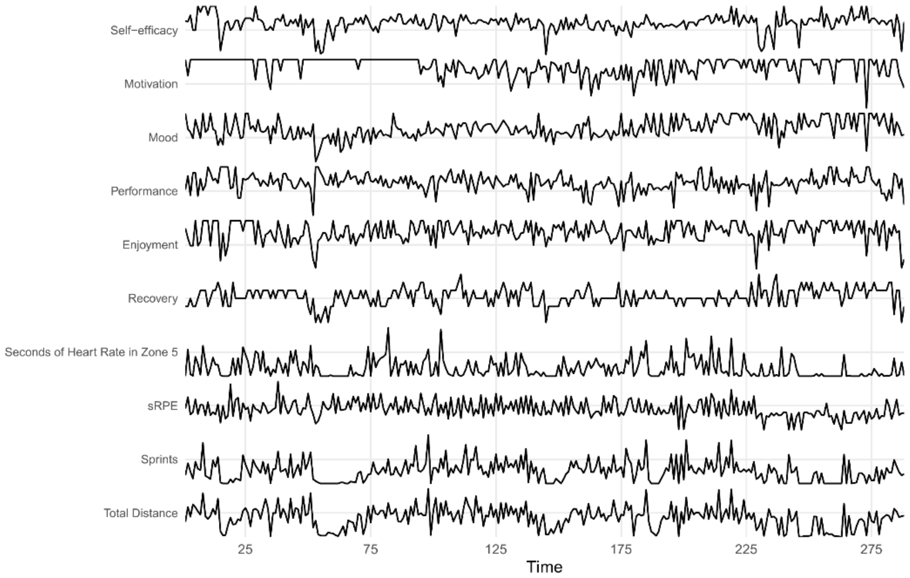

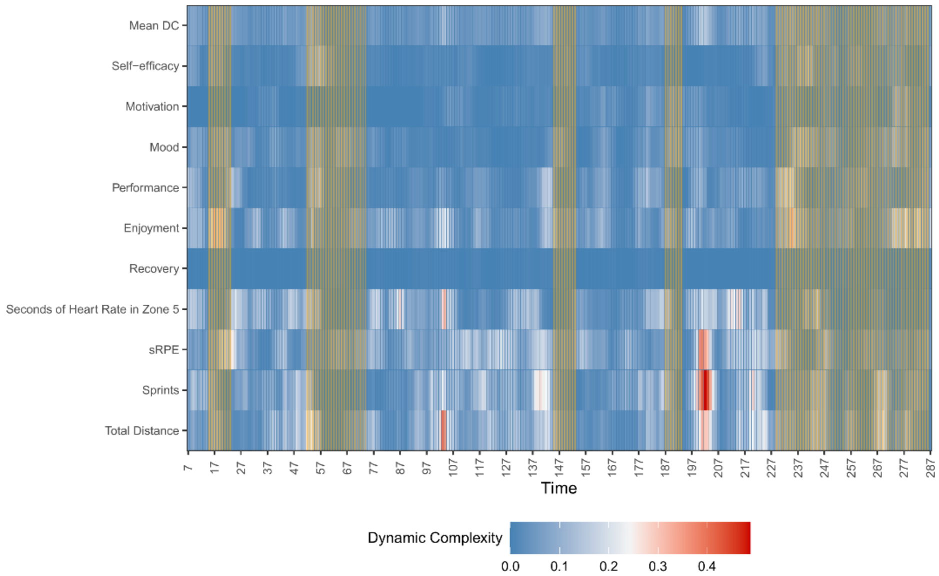

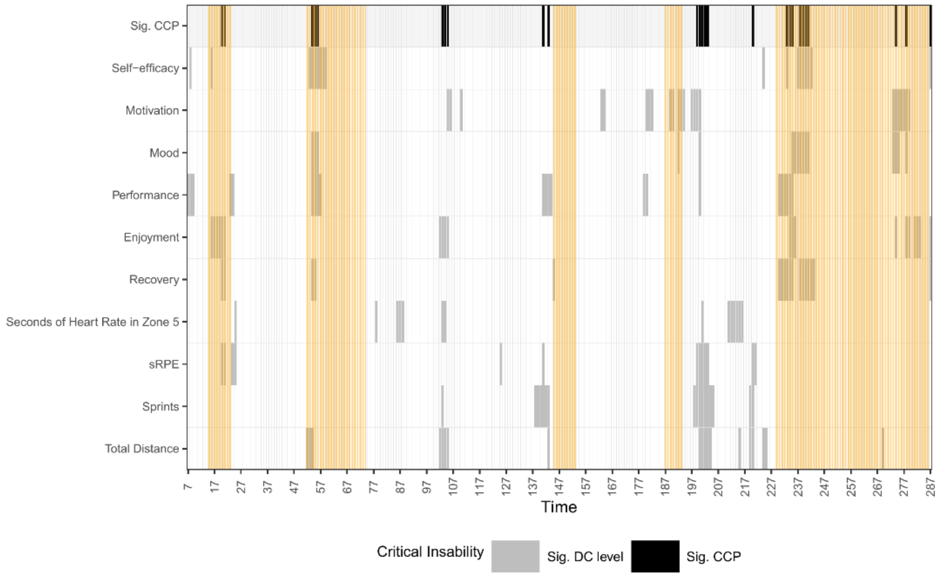

Supplementary Figure 15

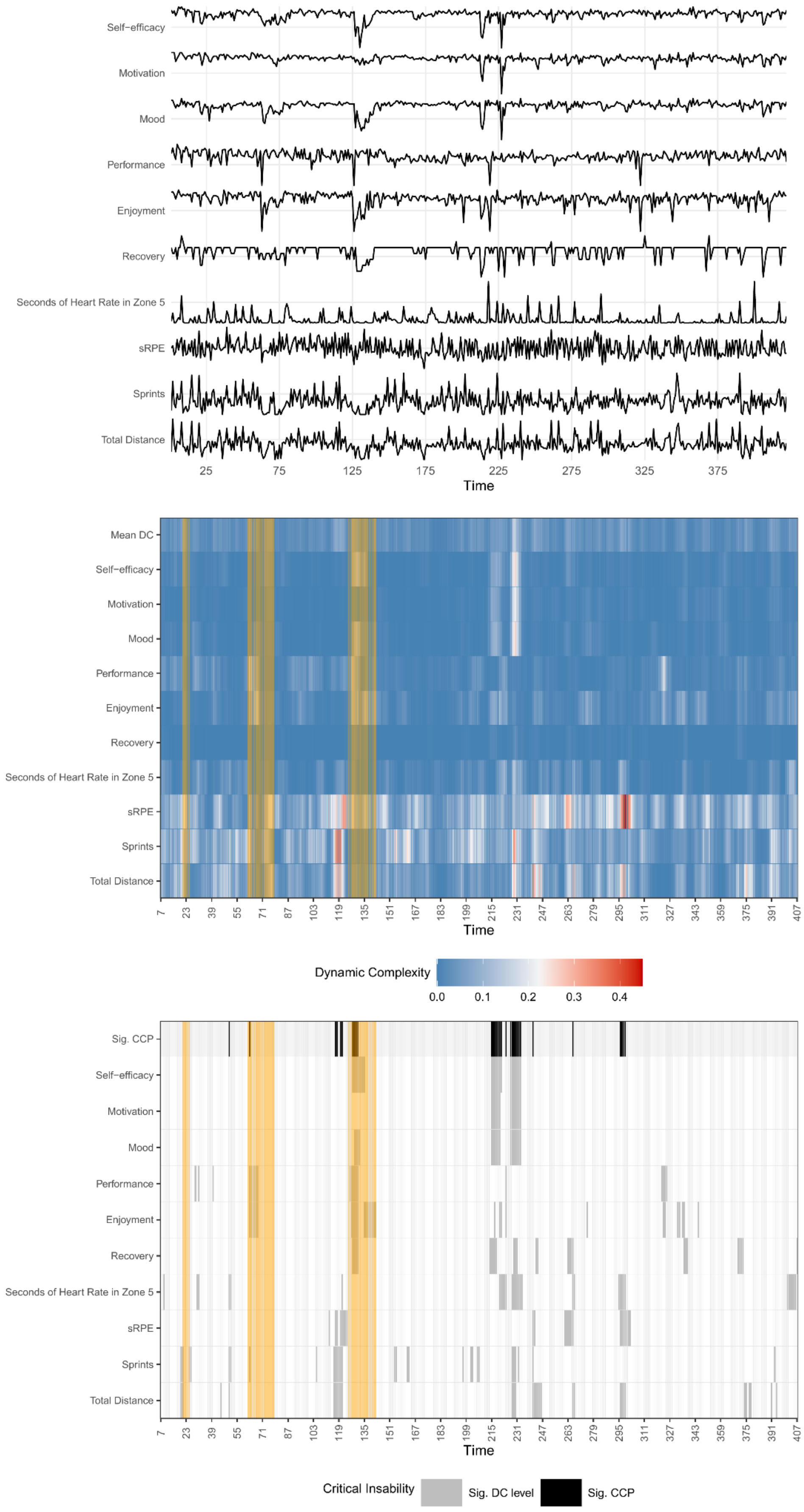

Supplementary Figure 16

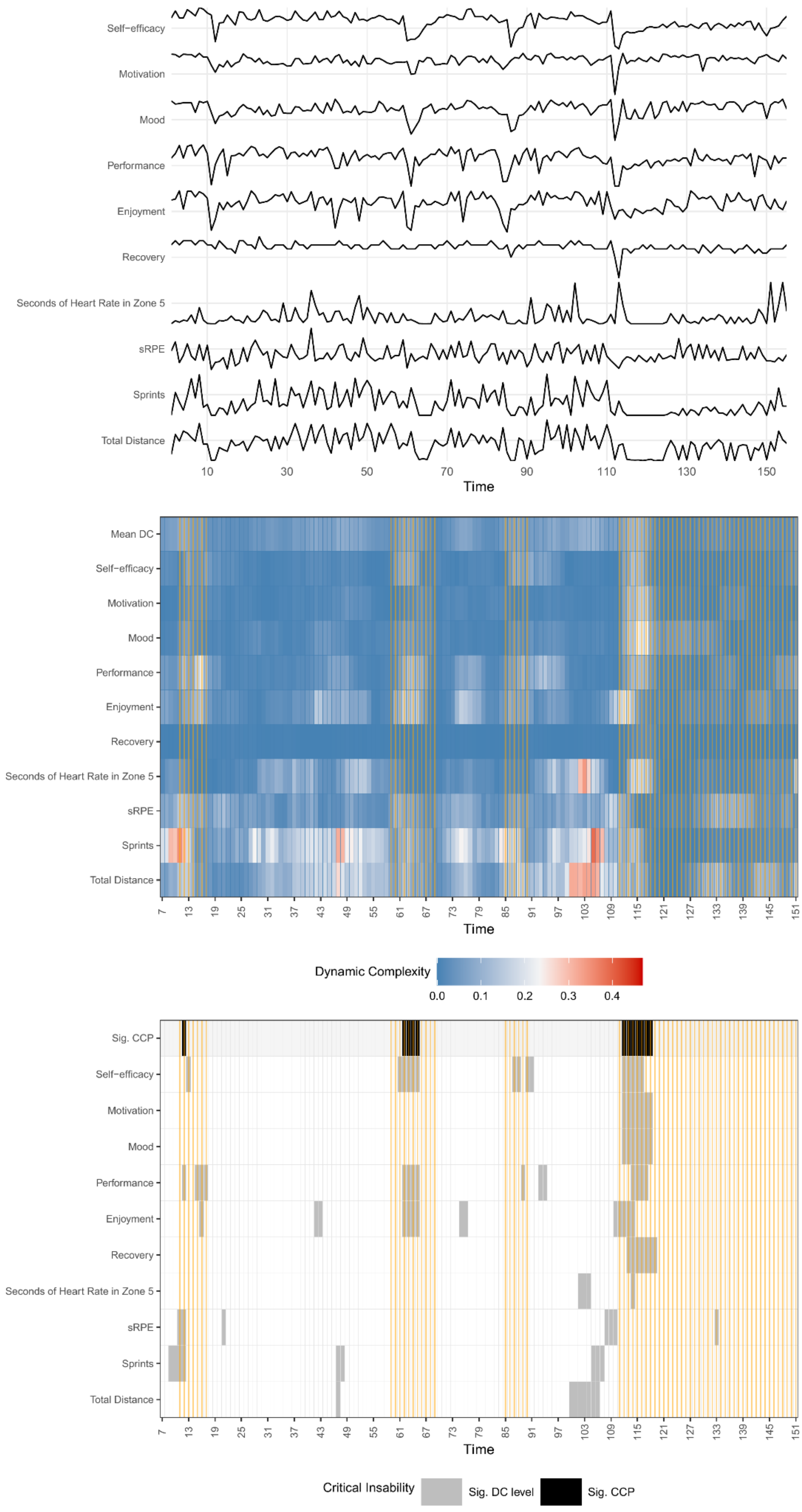

Supplementary Figure 17

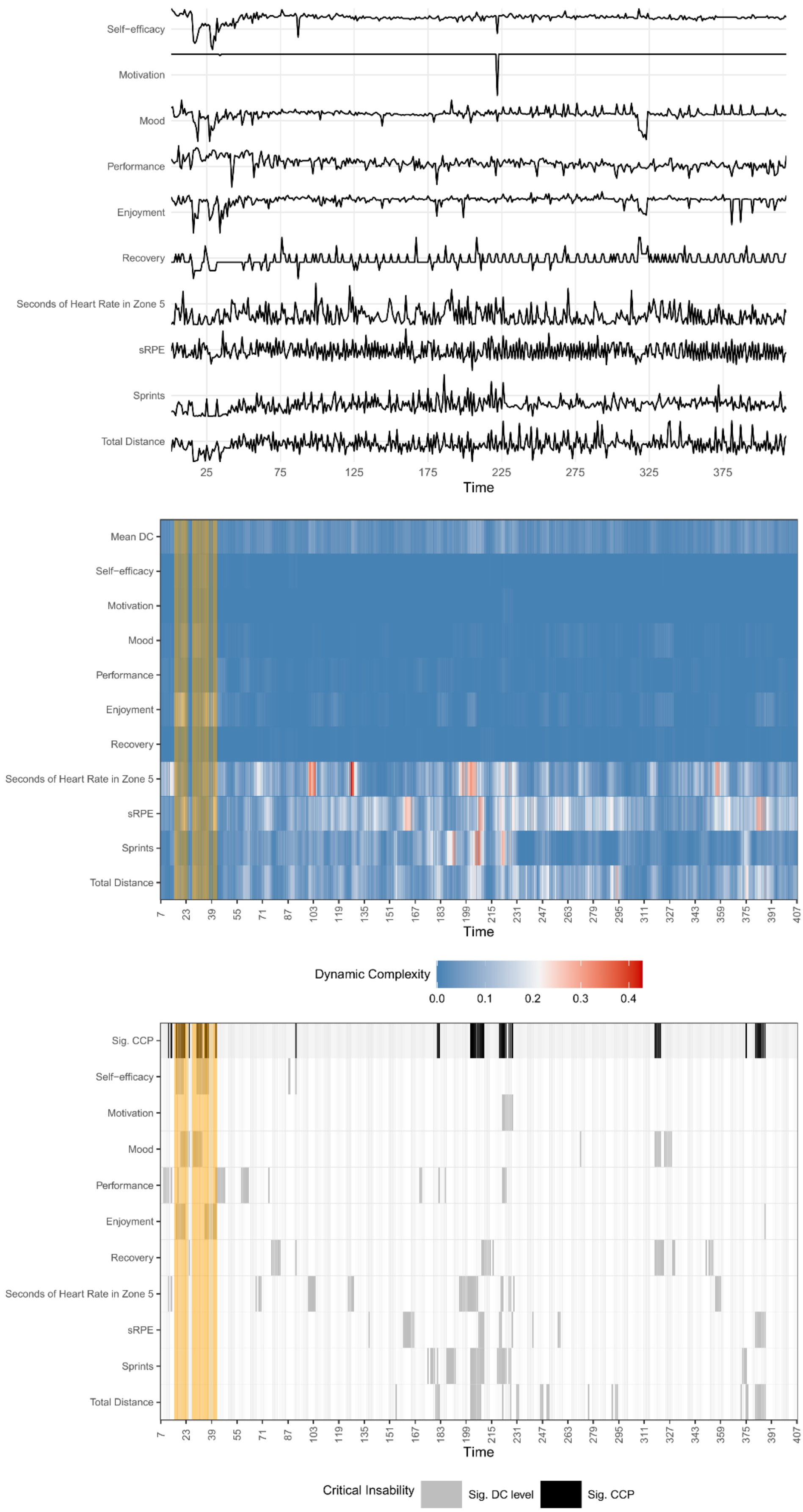

Supplementary Figure 18

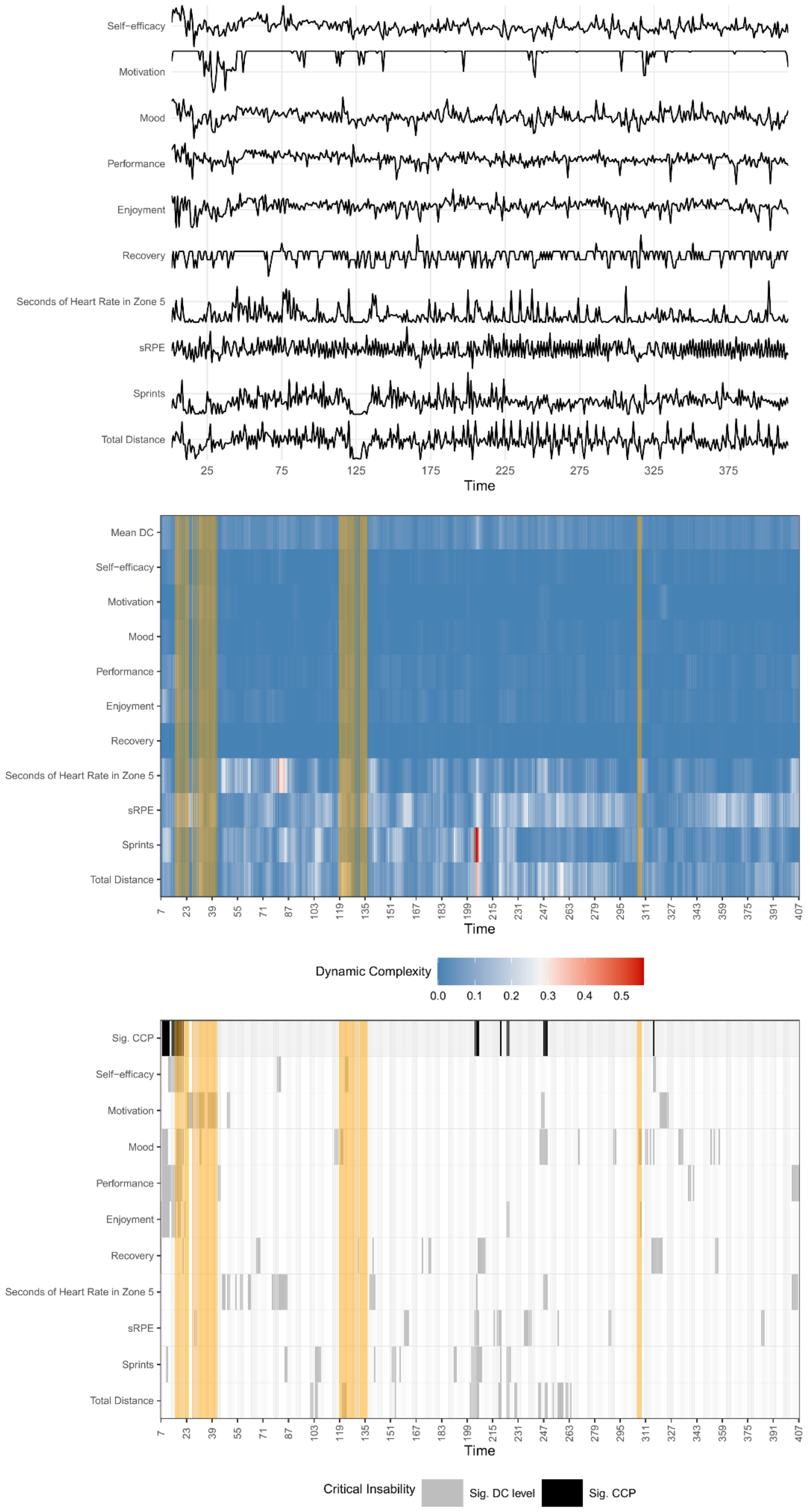

Supplementary Figure 19

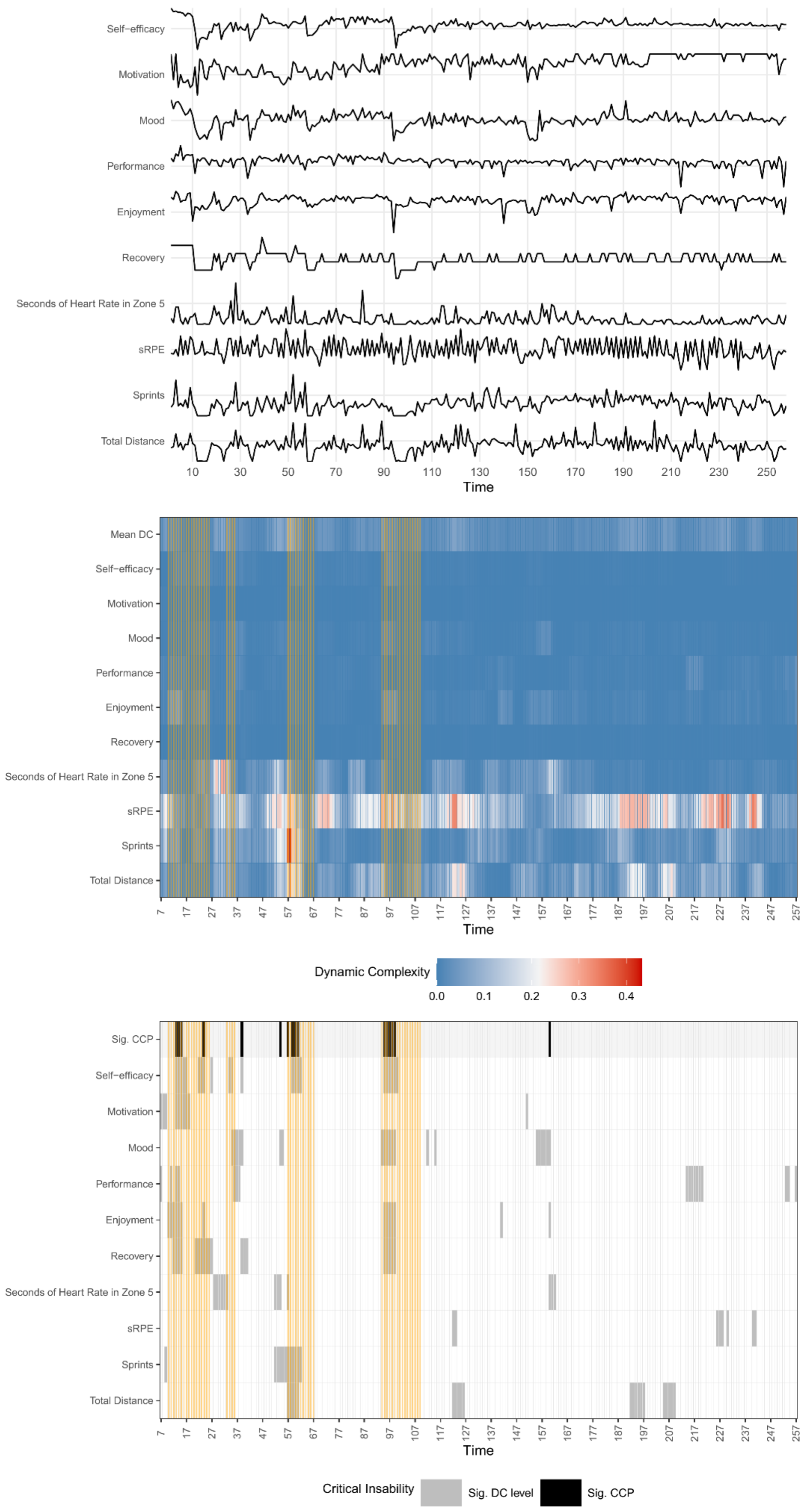

Supplementary Figure 20

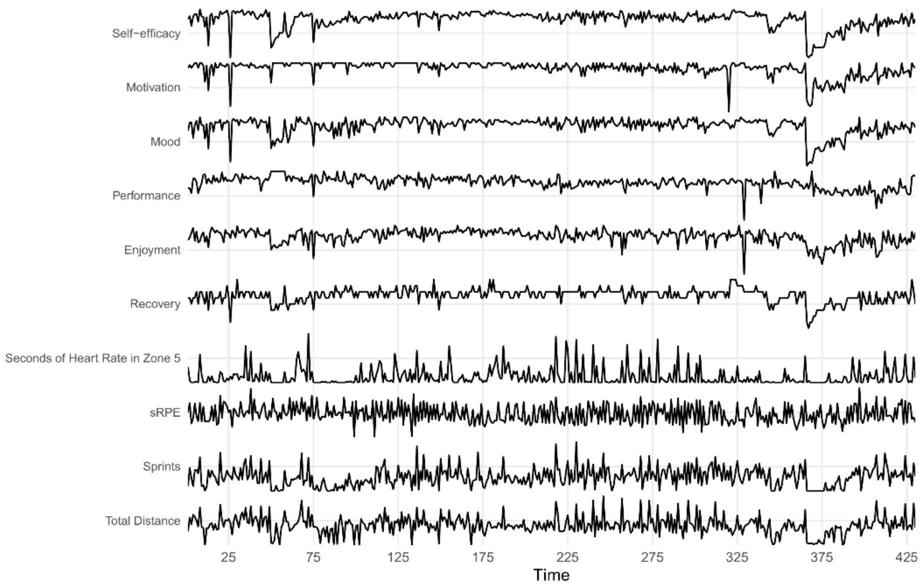

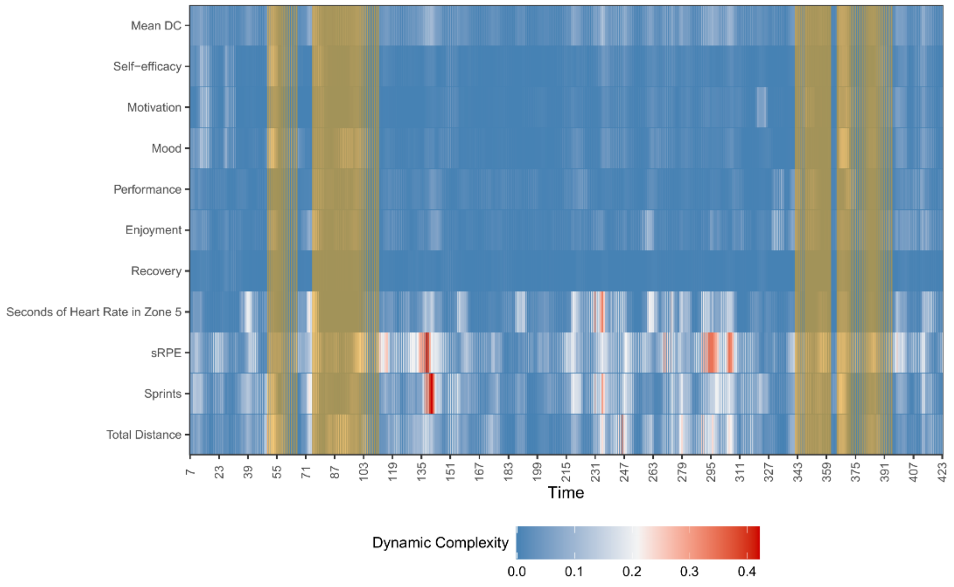

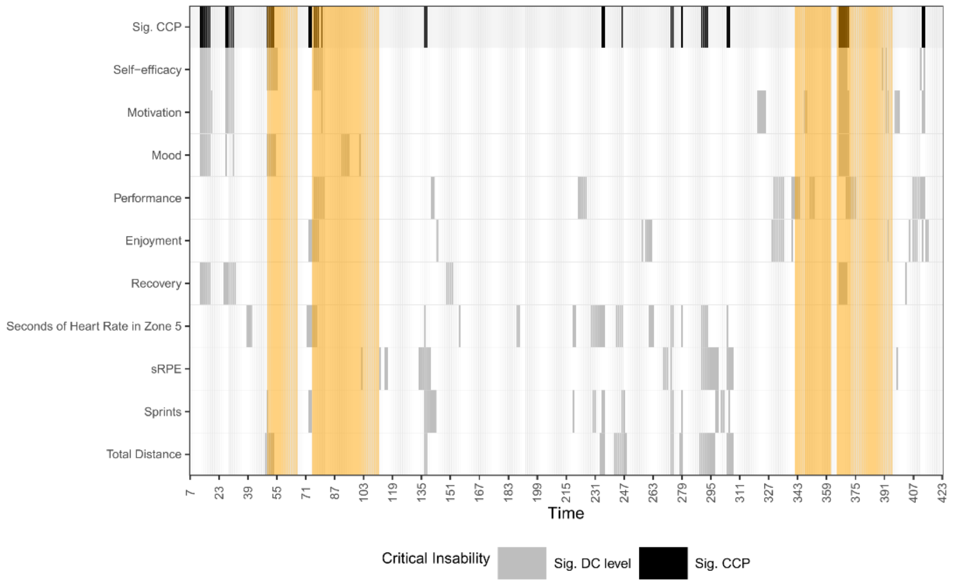

Supplementary Figure 21

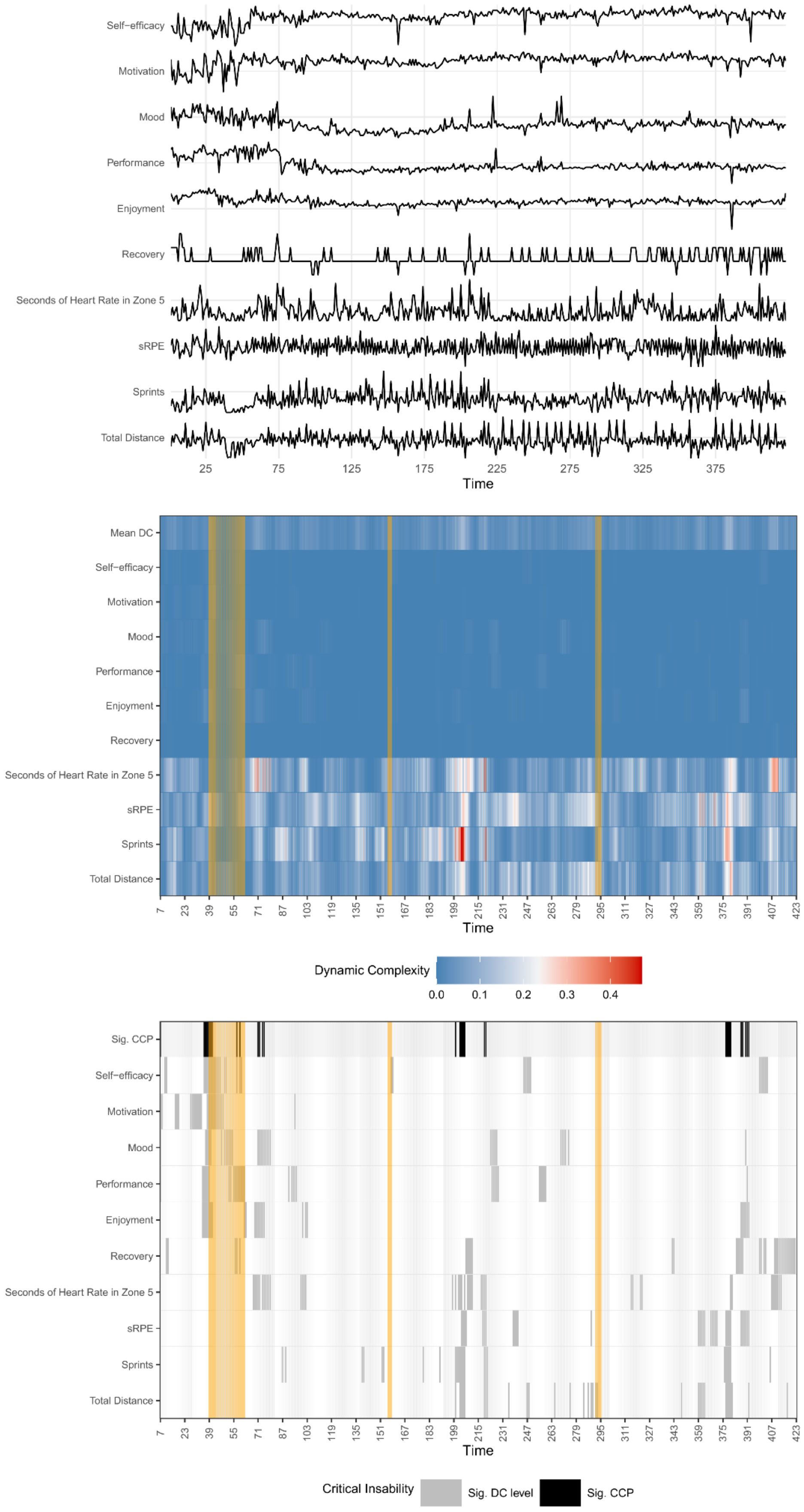

Supplementary Figure 22

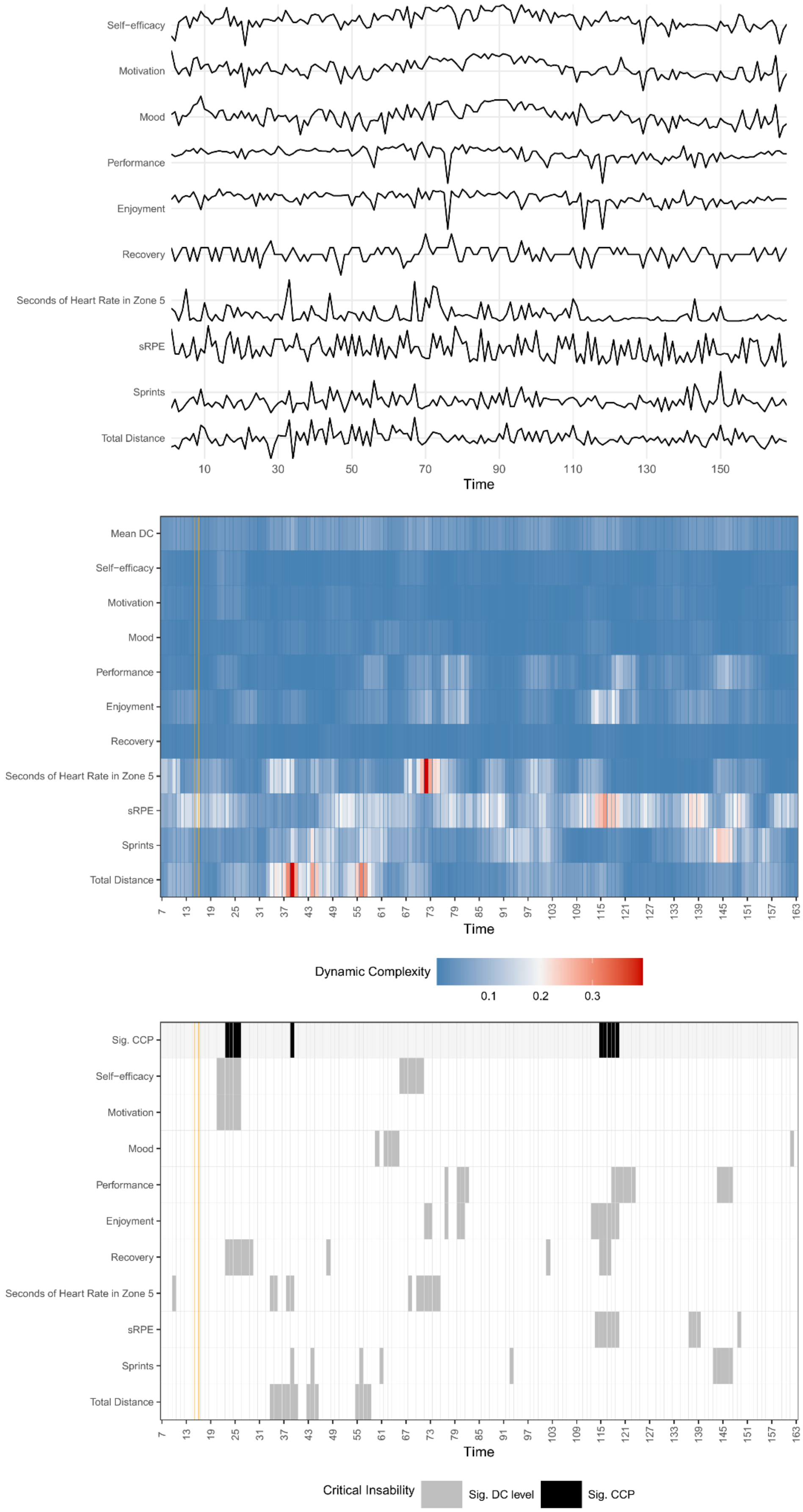

Supplementary Figure 23

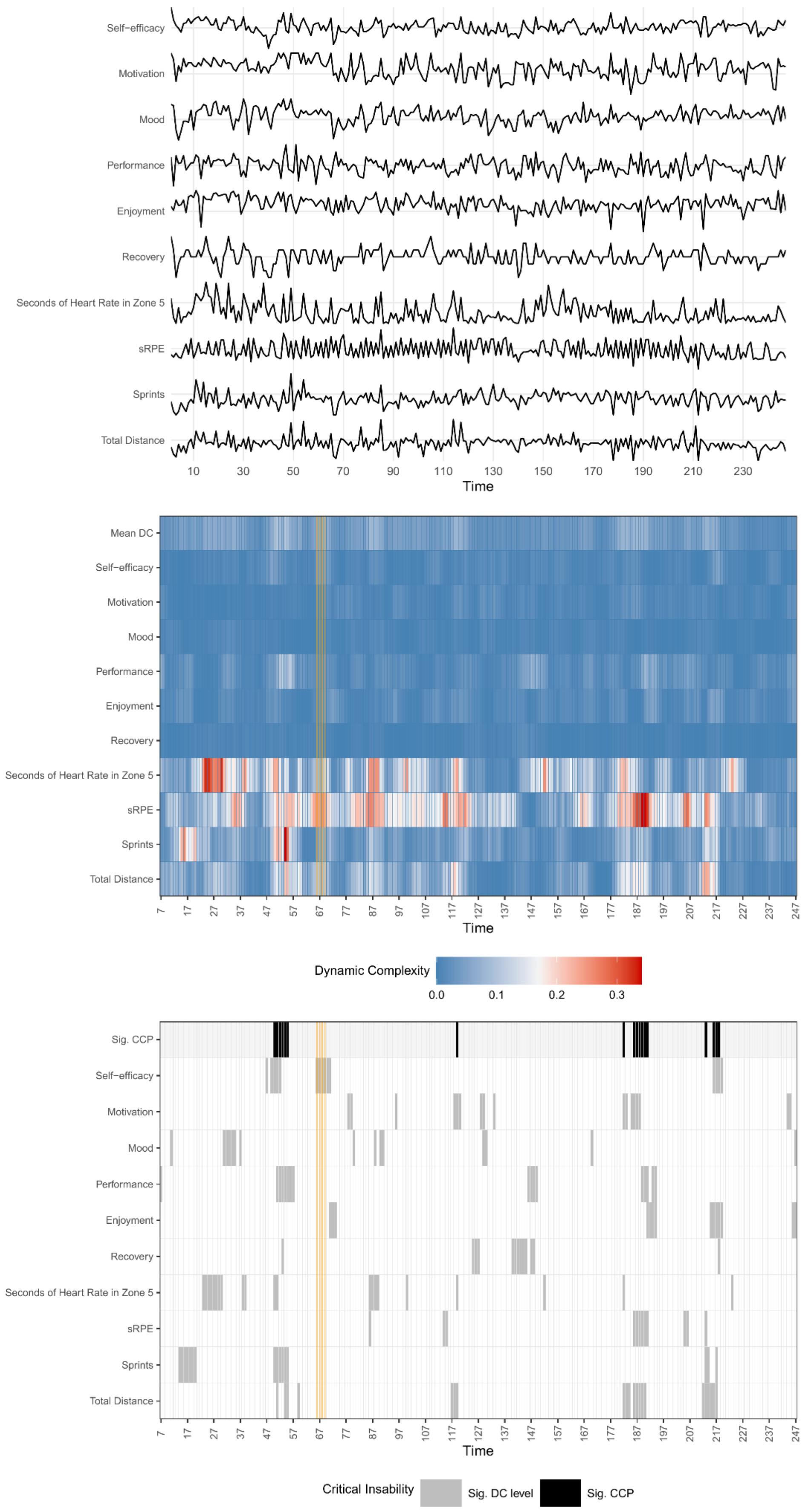

More Recommendations for Future Research

In our analysis, the detection of CCPs does not occur cumulatively, meaning that on any given day, such as day seven, it considers only the data available up to that point, i.e., seven days. Instead, it evaluates the entire time series that is available to the researcher at this moment. Adopting a cumulative approach would be more appropriate as it better reflects how the analysis would operate in real-time. Further, taking the injury history into account as well as investigating the dynamic evolvement of CCPs before injuries instead of looking at the single and static significant peaks could provide even more insights into the dynamics of how injuries occur. For instance, in Figure 2 of the main article, a warning signal appeared in the six data points before an injury. However, before data point seven, many more warning signals occurred. Hence, it would be interesting to investigate whether the dynamics of the warning signals provide valuable insights. Or, future investigations could study whether clustering CCPs that are close to each other in time (and then counted as one) could improve the explanatory performance of the model.
